# Do clinical guidelines support person-centred care for women affected by dementia: A content analysis

**DOI:** 10.1177/14713012241244982

**Published:** 2024-04-03

**Authors:** Nevetda Gengeswaran, Alec Brandwood, Natalie N Anderson, Jessica U Ramlakhan, Anna R Gagliardi

**Affiliations:** Toronto General Hospital Research Institute, 7989 University Health Network, Canada

**Keywords:** dementia, person-centred care, women’s health, clinical guidelines, content analysis

## Abstract

**Background:**

Dementia disproportionately affects women including persons living with dementia and caregivers. Person-centered care, rather than disease-focused, is recommended to improve care for affected persons including caregivers. General practitioners play a central role in dementia care but find it challenging due to inadequate training. The study aimed to assess if and how dementia guidelines provide clinicians with guidance on person-centred care for women affected by dementia.

**Methods:**

We searched for publicly available English-language guidelines on the overall management of dementia in MEDLINE, EMBASE and the Guidelines International Network repository. We employed deductive and summative content analysis, and categorized person-centered care guideline content based on established frameworks, and conveyed our results using summary statistics, text, and tables.

**Results:**

We reviewed 15 guidelines published from 2006 to 2020 in eight countries. Few (4, 23%) involved persons living with dementia or caregivers in guideline development. Regarding general person-centred care, guidelines mostly addressed the domains of exchange information (93%), share decisions (93%), enable self-management (93%) and address emotions (87%), while few offered content on manage uncertainty (33%) or foster a healing relationship (13%). Regarding dementia-specific person-centred care, most guidelines addressed intersectionality (tailoring care for diverse characteristics) (80%), but few included content on the domains of quality of life (67%), dignity (53%) or sex/gender issues (20%). Even when mentioned, the guidance was typically brief. We identified 32 general and 18 dementia-specific strategies to achieve person-centered care by compiling information from these guidelines.

**Conclusions:**

This study identified inconsistent and insufficient guideline content on person-centred care for women with dementia. Compiled strategies for achieving person-centred care could be used by developers to enhance existing and future dementia guidelines; and inform the development of policies or programs, education, tools for clinicians, and quality improvement measures for evaluating dementia care. Future research is crucial for promoting person-centred dementia care for women living with dementia.

## Background

Dementia is an increasingly prevalent condition characterized by gradual cognitive decline that impedes daily functioning (Canadian Academy of Health Sciences [CAHS], 2019; Gerlach & Kales, 2018; Tisher & Salardini, 2019). Its impact on physical, cognitive and mental health is often devastating for both persons living with dementia and their caregivers. Dementia is the second largest cause of disability for older persons and the seventh leading cause of death, and by 2030, it is estimated that 78 million people will have dementia (Prince et al., 2015).

Dementia disproportionately affects women. In 2021, 65% of total deaths due to dementia were in women (Prince et al., 2015), and based on 2019 data, the greater female:male ratio pattern was expected to continue to 2050 (Global Burden of Disease 2019 Dementia Forecasting Collaboration, 2022). Women face inequitable access to dementia care and support, often due to factors such as younger age of onset, delayed diagnosis of dementia, and socioeconomic status (Sourial et al., 2020). In a sample of 318,350 community-dwelling adults aged 65 and older, women had a higher occurrence of emergency department visits, lower continuity of care and longer discharge delays (Sourial et al., 2020). Furthermore, as most persons living with dementia reside at home, caregiving largely falls upon women, with wives more likely to support husbands than vice-versa, and daughters more likely to care for parents compared to sons (Bott et al., 2016), impacting caregiving women’s employment, health and well-being.

Despite the disproportionate and inequitable impact on women (i.e., persons who identify as women), limited public health or health system guidance is available on how to support women affected by dementia, who include persons living with dementia and women who are their caregivers (Bartlett et al., 2018). For example, analysis of national dementia strategies in 29 countries found they did not address sex or gender issues, thus offering no insight to leaders for health system planning (Chow et al., 2018). A scoping review on what constitutes high-quality dementia care in home, community or outpatient settings identified only 22 studies published from 2001 to 2019 (Marulappa et al., 2022). Most studies involved clinicians, either exploring barriers of dementia care (e.g., knowledge or attitudes about dementia, perceived lack of control or time) or evaluating the impact of educational strategies to improve clinician knowledge about dementia care (Marulappa et al., 2022). Three studies evaluated interventions targeted at persons living with dementia or caregivers, but did not report findings by sex or gender, thus offering no evidence to inform public health or health system policies on how to tailor and support dementia care for affected women (Marulappa et al., 2022).

Research shows that the needs of the growing number of persons living with dementia and their caregivers are not being met (Clarkson et al., 2018; Hinton et al., 2007; Morrisby et al., 2018; Yaffe et al., 2008). General practitioners, who play a central role in diagnosing and caring for those affected by dementia, report they lack training in dementia care, find it to be complex and challenging, and note a lack of dementia care guidance and support for primary care providers (Bourque & Foley, 2020; Koch et al., 2010; Mansfield et al., 2019). Furthermore, physicians lack training and knowledge in women’s health, which extends beyond reproductive health to conditions across the lifespan; and in person-centred care (Anderson & Gagliardi, 2021a, 2021b), which involves patients, family and clinicians working together in tailoring care to individual health needs, life circumstances and personal preferences (Epstein & Street, 2011; Institute of Medicine (US) Committee on Quality of Health Care in America., 2001). Person-centred care optimizes patient care experiences and clinical outcomes (Doyle et al., 2013; Rathert et al., 2013). It long been advocated in the context of dementia rather than disease-centred care (Fazio et al., 2018; Kim & Park, 2017), and stands to reduce gendered inequities in access to and quality of dementia care because it focuses on personalizing care (Erol et al., 2015).

Clinicians often refer to clinical practice guidelines, which are decision-making tools that inform the approaches and treatments to offer when caring for individuals with specific conditions. Guidelines consolidate and synthesize scientific evidence, and offer recommendations for care delivery that can enhance patient-important and clinical outcomes (Shekelle et al., 2012). While dementia guidelines have been appraised for methodological quality of their development and for consistency in clinical recommendations across guidelines (Azermai et al., 2012; Ngo & Holroyd-Leduc, 2015), no prior research has assessed whether or how dementia guidelines support person-centred care, particularly for women affected by dementia.

Given little guidance in policy or research on how to optimize care and support for women affected by dementia (Bartlett et al., 2018; Chow et al., 2018; Marulappa et al., 2022), and the potential role of guidelines in doing so (Shekelle et al., 2012), the purpose of this study was to assess if and how dementia guidelines address person-centred care for women affected by dementia. The specific objective was to assess dementia guidelines for content on the care and support of women with diverse characteristics who have dementia or are caring for persons living with dementia. Exemplar content could be widely emulated by developers of dementia guidelines, and those guidelines could inform public and health system dementia policies and programs. Identifying gaps in content could help guidelines developers strengthen the relevance of dementia guidelines so that they better support clinicians in achieving equitable, person-centred dementia care.

## Methods

### Approach

We used content analysis to assess the content of international dementia guidelines (Elo & Kyngäs, 2008; Hsieh & Shannon, 2005). This approach employs both deductive and summative content analysis procedures to first organize and describe content in categories (deductive), and then count and compare categories across documents (summative). In the absence of reporting criteria specific to content analyses, to enhance rigor, we complied with the Standards for Reporting Qualitative Research (O’Brien et al., 2014). We did not obtain ethical approval because the study did not involve human participants from whom we needed to obtain informed consent.

### Eligibility criteria

We included English-language guidelines on the overall management of dementia, referring to two or more of screening, diagnosis, any form of therapy or supportive care for Alzheimer’s disease or other major forms of dementia (e.g., Parkinson’s, Lewy Body disease, frontotemporal disease). We chose to include guidelines that addressed overall management so that the sample would include guidelines with uniform characteristics, given the many guidelines related to dementia on a wide array of specialized topics. We included guidelines published from 2000 onwards to capture guidelines the reflected up-to-date underlying clinical evidence, and because it coincided with publication of a landmark report that recognized person-centred care, after which time guideline developers may have been aware of this concept (Institute of Medicine (US) Committee on Quality of Health Care in America, 2001). Guidelines referred to publicly-available new, updated or adapted/adopted documents developed using standardized methods including a systematic review of evidence, assessment of the benefits and harms of alternative care options, and synthesis of evidence into one or more recommendations. Eligible guidelines were developed in any country by non-profit organizations such as professional societies, academic institutions, government agencies, disease-specific foundations, or quality improvement/monitoring agencies. Guidelines were not eligible if they did not employ systematic methods to formulate evidence-based recommendations (e.g., consensus statements), or if they focused only on prevention or end-of-life care for dementia, or on topics relevant to ageing but without a focus on dementia.

### Searching and screening

We searched for guidelines in MEDLINE and EMBASE, the largest indexed databases of published medical research most likely to include dementia guidelines, using [(dementia or Alzheimer disease) AND (practice guidelines as topic or guidelines as topic) OR (publication type: guideline or practice guideline)] in December 2021 and updated the search in December 2022. We also searched a comprehensive repository of international guidelines maintained by the Guidelines International Network with the keyword dementia. Searches were conducted and compiled by NG, AB (both trainees) and NNA (Master-trained Research Associate). To pilot test screening, NG, AB, NNA and ARG (PhD-trained investigator) independently screened the first 50 titles/abstracts against eligibility criteria, then met to discuss and resolve discrepancies. This was repeated twice until selection of potentially eligible items was congruent. NG and AB retrieved full-text guidelines and assessed their eligibility concurrent with data extraction, and NNA and ARG independently resolved uncertainties.

### Data extraction

We extracted data on guideline characteristics and content related to person-centred care. Guideline characteristics included year of publication, developer organization, type of developer (professional society, government, academic group, charity), country, development methods (if persons living with dementia/caregivers involved in development), dementia cause (Alzheimer’s only or other causes) and dementia stage (mild, moderate, advanced). For general aspects of person-centred care, data included content relevant to a framework generated by McCormack et al. (2011). We chose this framework to guide data extraction because it was rigorously-developed based on a literature review, observation of clinical encounters, and input from patients and healthcare professionals (Scholl et al., 2014). With 31 elements across six domains, it is more comprehensive than other person-centred care frameworks (Scholl et al., 2014). While originally developed in the cancer context, its relevance to women with a range of clinical conditions was validated in other research (Filler et al., 2020; Gagliardi et al., 2020; Nyhof et al., 2020). The six domains include foster a healing relationship, exchange information, address emotions and concerns, manage uncertainties, share decisions and enable self-management. For dementia-specific aspects of person-centred care, there is no established framework or universal agreement. However, we derived key themes from a synthesis of published research that described person-centred dementia care: focus on dignity, referring to value and respect for self-hood or person-hood; and quality of life, referring to self-determination, purposeful living and a positive social environment (Fazio et al., 2018). In addition to general and dementia-specific aspects of person-centred care, we also extracted data on mention of sex, gender or intersectional factors (e.g., age, ethnicity, culture, education, employment, marital/partnership status, ability, sexual orientation, urbanity/rurality), which clinicians must consider when tailoring dementia care to individuals. To pilot test data extraction, NG, AB, NNA and ARG independently extracted data from two guidelines, where text as it appeared in the guideline was copied and pasted into a table organized according to general and dementia-specific aspects of person-centred care. They met to discuss and resolve discrepancies. This process was repeated once more, at which time data extraction was congruent. Thereafter, NG and AB extracted data from remaining guidelines. All data were reviewed independently by NNA and ARG.

### Data analysis

We used summary statistics to report guideline characteristics, and the number of guidelines that addressed general and dementia-specific person-centred care domains. We used tables and text to highlight the degree to which guidelines addressed these domains. We synthesized strategies recommended across guidelines for achieving both general and dementia-specific person-centred care.

## Results

### Search results

**Figure 1.**
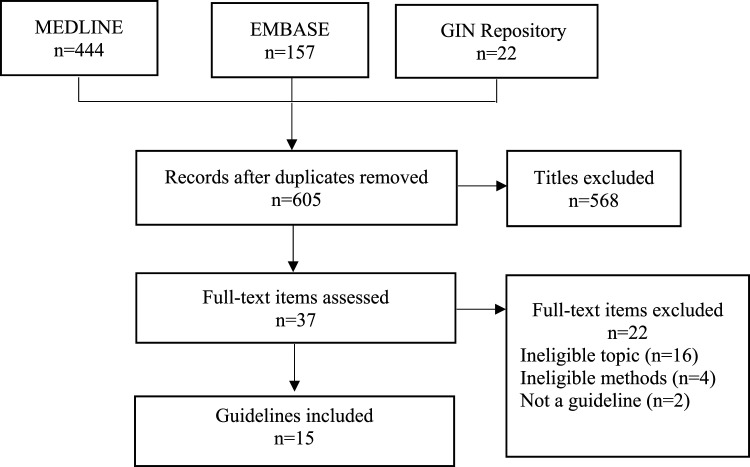
PRISMA diagram. Flow chart of search results and guidelines included.

The search yielded 623 results, of which 605 were unique. After title and abstract screening, we excluded 568 items and 37 remained. After full text screening, we excluded 22 items due to clinical topic (*n* = 16), methodology (*n* = 4) and publication type (*n* = 2). Overall, we included 15 guidelines in this study (Figure 1).

### Guideline characteristics

Additional File 1 provides data extracted from included guidelines. Guidelines were published between 2006 and 2020 in the United States (4, 26.7%), Canada (3, 20.0%), Australia (2, 13.3%) and United Kingdom (2, 13.3%), and 1 (6.7%) each in the Czech Republic, Malaysia, Singapore and Spain (Table 1). Most guidelines were developed by professional societies (9, 60.0%). With respect to methods, only 4 (23.3%) involved persons living with dementia or caregivers in guideline development. All guidelines addressed the full spectrum of clinical management for Alzheimer’s disease. Fewer also addressed Lewy Body dementia (12, 80.0%), Vascular dementia (12, 80.0%) and Fronto-temporal dementia (9, 60.0%). All guidelines focused on mild or early stages of dementia, and all but one (14, 93.3%) also included moderate and advanced dementia.

**Table 1.** Characteristics of included guidelines.

Characteristic	Category	Guidelines (*n*, % of 15)
Country	United States	4 (26.7)
	Canada	3 (20.0)
	Australia	2 (13.3)
	United Kingdom	2 (13.3)
	Other	4 (26.7)
Developer type	Professional society	9 (60.0)
	Government	5 (33.3)
	Academic	1 (6.7)
Persons living with dementia/caregivers involved in guideline development	No	11 (73.3)
	Yes	4 (26.7)
Care stage	Overall management	15 (100.0)
	Treatment	15 (100.0)
	Diagnosis	13 (86.7)
Dementia cause	Alzheimer’s disease	15 (100.0)
	Lewy body dementia	12 (80.0)
	Vascular dementia	12 (80.0)
	Fronto-temporal lobe dementia	9 (60.0)
Dementia stage	Mild	15 (100.0)
	Moderate	14 (93.3)
	Advanced	14 (93.3)

### General aspects of person-centred care

Additional File 2 provides all data extracted from included guidelines pertaining to general aspects of person-centred care. Table 2 summarizes domains included in each guideline. Two guidelines addressed all six domains (Abbey et al., 2008; Ministry of Health, Malaysia, 2009). Guidelines most commonly addressed the domains of exchange information (14, 93.3%), share decisions (14, 93.3%), enable self-management (14, 93.3%) and address emotions (13, 86.7%). Few guidelines addressed the domains of manage uncertainty (5, 33.3%) and foster a healing relationship (2, 13.3%).

**Table 2.** General aspects of person-centred care described in included guidelines.

Guideline	General person-centred care domains (*n*, % of 15)					
	Foster the relationship	Exchange information	Address emotions	Manage uncertainty	Share decisions	Enable self-management
Ismail et al., 2020, Canada	--	+	-	+	+	+
National Institute for Health and Care Excellence, 2018, United Kingdom	--	+	+	--	+	+
Toward Optimum Practice, 2017, Canada	--	--	+	--	+	+
Guideline Adaptation Committee, 2016, Australia	--	+	+	--	+	+
Government of British Colombia, 2016, Canada)	--	+	+	--	+	+
Nagaendran et al., 2013, Singapore	--	+	+	--	+	+
Hort et al., 2010, Czech Republic	--	+	+	+	+	--
Ministry of Health Social Services and Equality, 2010, Spain	--	+	+	--	+	+
Ministry of Health, 2009, Malaysia	--	+	+	--	+	+
Amante et al., 2012, United States	--	+	+	+	+	+
Abbey et al., 2008, Australia	--	+	+	--	+	+
California Workgroup on Guidelines for Alzheimer’s Disease Management, 2008, United States	+	+	+	+	+	+
Rabins et al., 2007, United States	+	+	+	+	+	+
Fillit et al., 2006, United States	--	+	--	--	--	+
Scottish Intercollegiate Guidelines Network, 2006, Scotland	--	+	+	--	+	+
Total	2 (13.3)	14 (93.3)	13 (86.7)	5 (33.3)	14 (93.3)	14 (93.3)

Table 3 provides examples of general person-centred care content extracted from included guidelines and 32 key strategies offered across guidelines for each domain. With respect to foster a healing relationship, two guidelines provided scant insight despite recommending forming an alliance with persons living with dementia and caregivers. Regarding exchange information, some guidelines stated the need for good communication but provided little additional guidance, while others offered more detail on how to achieve good communication. Across all guidelines, 12 strategies emerged; for example, communication should be sensitive and empathic, assess and document a complete history, engage caregivers in such discussions, assess changes over time, provide information in verbal and print format, and provide information that is relevant to stage of condition. However, it is notable that most guidelines that addressed this domain focused on one-way communication of information from providers to persons living with dementia and caregivers, with little to no emphasis on two-way communication beyond clinical history-taking. While most guidelines included some content for address emotions, the level of detail varied across guidelines, with most focusing on dementia-related agitation or caregiver respite rather than how to help personx living with dementia and caregivers deal with the trauma of such a diagnosis. Similarly, the 3 strategies that emerged for this domain pertained to referral for support rather than providing support: routinely inquire about concerns, assess caregiver need for support, and connect persons living with dementia and caregivers with community supports or services. The few guidelines that addressed manage uncertainties included brief content pertaining to this domain regarding 3 strategies: set realistic expectations, reassure persons living with dementia and caregivers about working with them to manage issues as they arise, and routinely ask persons living with dementia and caregivers about specific concerns. Most guidelines detailed the need to share decisions, encapsulated in 6 strategies; for example, discuss the benefits and harms of treatment with persons living with dementia and caregivers, supplement discussion with information tools such as decision aids, and routinely revisit preferences and decisions regarding advanced care plans. Regarding enable self-management, most guidelines offered detailed insight on how to help persons living with dementia and caregivers to cope with dementia, captured by 7 strategies; for example: fully assess each person’s circumstances to create an individualized self-management plan, routinely monitor needs and adjust the plan as needed, describe coping strategies, and ensure that persons living with dementia and caregivers are aware of community supports and services.

**Table 3.** Examples of general person-centred care content in included guidelines.

PCC domain	Brief example	Detailed example	Recommended strategies
Foster healing relationship	Establishing and maintaining alliances with caregivers is critical for care of the Alzheimer’s disease patient (Ministry of Health, Malaysia, 2009).	Establish and maintain an alliance with the patient and the family. As with any psychiatric care, a solid therapeutic alliance is critical to the treatment of a patient with dementia. The care of a patient with dementia requires an alliance with the patient, as well as with the family and other caregivers. (Abbey et al., 2008)	• Form an alliance with persons living with dementia and caregivers
Exchange information	They should be given ample opportunity to ask questions and seek clarification from the doctor (National Institute for Health and Clinical Excellence, 2018).	Health and aged care professionals should discuss with the person any need for information to be shared. However, as the condition progresses and the person with dementia becomes more dependent on family or other carers, decisions about sharing information (with other health professionals or substitute decision makers) should be made in the context of the person’s capacity to make decisions. If information is to be shared, this should be done only if it is in the best interests of the person with dementia. Although a very small number of people may choose not to know the diagnosis, it is clear that the majority of people want to be informed and therefore, it is important that health professionals are honest and truthful when communicating the diagnosis to the person with dementia and those close to them. The diagnosis of dementia is never provided without earlier discussion about memory and thinking difficulties. Medical practitioners should discuss the possibility of dementia as a diagnosis during the process of assessment, which may take three to six months to achieve. Discussion of the diagnosis and its consequences may occur gradually over several visits to the medical practitioner, but should occur as early as practicable. Following a diagnosis of dementia, health and aged care professionals should, unless the person with dementia clearly indicates to the contrary, provide them and their carer(s) and family with written and verbal information in an accessible format about: The signs and symptoms of dementia, the course and prognosis of the condition, treatments, and sources of financial and legal advice, and advocacy. Health and aged care professionals should ensure that the person with dementia and his or her carer(s) and family are provided with written and verbal information regarding appropriate services available in the community. Any advice and information given should be recorded (Rabins et al., 2007).	• Be vigilant for cognitive symptoms reported by patients or caregivers
	Good communication between healthcare professionals, patients and carers is essential (Scottish Intercollegiate guidelines Network).		• Ask for concerns about memory or cognition
			• Engage caregivers in such discussions
			• Explore underlying beliefs and attitudes about dementia
			• Assess and document a complete history
			• Employ screening tools to supplement discussion
			• Assess changes over time; don’t rely on a single discussion or tool
			• Provide information that is relevant to stage of condition in both oral and written format
			• At diagnosis, ask for consent to share information and who
			• Respect the rights of those who do not want to know about their medical condition
			• Provide information about available community services
			• Communication should be sensitive and empathic
Address emotions	Assist people to maintain confidence and a positive self-esteem (Rabins et al., 2007).	Because family members are often responsible for implementing and monitoring treatment plans, their own attitudes and behaviors can have a profound effect on the patient, and they often need the treating physician’s compassion and concern. For these reasons, treatment is directed to the patient-caregiver system. Family members often feel overwhelmed by the combination of hard work and personal loss associated with caring for an individual with dementia. The caring and pragmatic attitude of the psychiatrist may provide critical support. This attitude may be expressed through thoughtful inquiries about current needs and how they are being met, advice about available sources of emotional and practical support, referrals to appropriate community resources, and supportive psychotherapy. Psychiatrists caring for patients with dementia should be vigilant for these conditions in caregivers, because they increase the risk of substandard care, neglect, or abuse of patients and are a sign that the caregivers themselves are in need of care. When a caregiver is in significant distress, his or her need for greater psychosocial support should be evaluated. If treatment is indicated, it can be provided (according to the preference of psychiatrist, patient, and caregiver) by the patient’s psychiatrist or through a referral to another mental health professional (Abbey et al., 2008)	• On each visit, ask about emerging concerns and need for support
			• Connect persons living with dementia and caregivers with community supports and respite services
			• Routinely assess caregiver well-being
Manage uncertainties	Realistic expectations for treatment effects and potential side effects should be discussed with the patient and caregivers (toward Optimum practice. 2017).	Delivering a diagnosis of dementia: Talking points	• Set realistic expectations regarding treatment effects
		• Eventually, dementia will cause a worsening in your ability to handle regular tasks, such as shopping, finances, and medications. But we’ll talk regularly, and we’ll manage that.	• Reassure persons living with dementia and caregivers of ongoing monitoring to manage issues as they arise
		• Dementia is a progressive condition with no cure, but we have treatments for symptoms. And proper care and planning can greatly alleviate the burden of dementia (Nagaendran et al., 2013).	• Ask persons living with dementia and caregivers about specific concerns
Share decisions	Before starting antipsychotics, discuss the benefits and harms with the person and their family members or carers (as appropriate) (Hort et al., 2010).	People have the right to be involved in discussions and make informed decisions about their care	• Consider preferences of persons living with dementia and caregivers
		Encourage and enable people living with dementia to give their own views and opinions about their care.	• Assess persons living with dementia decision-making capacity, and if needed, identify a surrogate
		Offer early and ongoing opportunities for people living with dementia and people involved in their care to discuss:	• Routinely review preferences and decisions (advance care plans)
		• Benefits of planning ahead	• Engage them in shared decision-making
		• Lasting power of attorney (for health and welfare decisions and property and financial affairs decisions)	• Discuss benefits and harms of treatment
		• An advance statement about their wishes, preferences, beliefs and values regarding their future care	• Supplement discussion with information tools, decision aids
		• Advance decisions to refuse treatment	
		• Their preferences for place of care and place of death.	
		Explain that they will be given chances to review and change any advance statements and decisions they have made. At each care review, offer people the chance to review and change any advance statements and decisions they have made (Hort et al., 2010)	
Enable self-management	Alzheimer’s disease care management should include referrals for discussions of advanced directives, proxy assignment, and durable power of attorney, as well as financial planning for long-term care and medical assistance, as needed (Scottish Intercollegiate Guidelines Network, 2006).	Carers and families of people with dementia should be supported to build resilience and maintain overall health and fitness. Promote and maintain functional and social independence of people with dementia in community and residential care settings. Interventions should address activities of daily living that maximize independence, function and engagement. Intervention should include:	• Fully assess each person’s circumstances to develop an individualized plan
		• Consistency of care staff	• Periodically monitor needs and adjust care plan
		• Stability in living environment	• Provide advice verbally and in print format
		• Flexibility to accommodate fluctuating abilities	• Offer caregivers psycho-education and skills training
		• Support for people with dementia and their carer(s) and families to participate in tailored activities that are meaningful and enjoyable	• Monitor medication management and safety risks to offer aids as needed
		• Assessment and intervention, involving the carer(s) and family wherever possible, to promote independent self-care skills and prevent excess disability, in particular supporting the person with dementia to retain continence	• Offer coping strategies; for example, advise caregivers to redirect and distract person living with dementia, and remove triggers to ease agitation
		To assist the carer(s) and family help the person with dementia who is experiencing behavioural and psychological symptoms of dementia, carer(s) and family should be offered interventions which involve:	• Ensure that person living with dementia and caregivers are aware of community supports
		• Carer skills training in managing symptoms and communicating effectively with the person with dementia	
		• Meaningful activity planning	
		• Environmental redesign and modification to improve safety and enjoyment	
		• Problem solving and management planning (Rabins et al., 2007).	

### Dementia-specific aspects of person-centred care

Additional File 3 provides data extracted from included guidelines pertaining to dementia-specific aspects of person-centred care. Table 4 summarizes domains included in each guideline. One guidelines addressed all 4 domains (Abbey et al., 2008) Most guidelines addressed the domain of intersectionality (12, 80.0%). Fewer guidelines addressed quality of life (10, 66.7%) and dignity (8, 53.3%), and very few addressed issues of sex or gender (3, 20.0%).

**Table 4.** Dementia-specific aspects of person-centred care described in included guidelines.

Guideline	Sex/gender and dementia-specific person-centred care domains (*n*, % of 15)			
	Sex/Gender	Intersectionality	Dignity	Quality of life
Ismail et al., 2020, Canada	--	--	--	--
National Institute for Health and Care Excellence, 2018, United Kingdom	+	+	--	+
Toward Optimum Practice, 2017, Canada	--	+	--	--
Guideline Adaptation Committee, 2016, Australia	--	+	+	+
Government of British Colombia, 2016, Canada)	--	+	+	--
Nagaendran et al., 2013, Singapore	--	+	+	+
Hort et al., 2010, Czech Republic	--	+	--	--
Ministry of Health Social Services and Equality, 2010, Spain	--	--	+	+
Ministry of Health, 2009, Malaysia	--	+	--	+
Amante et al., 2012, United States	--	+	+	+
Abbey et al., 2008, Australia	--	+	+	+
California Workgroup on Guidelines for Alzheimer’s Disease Management, 2008, United States	--	+	+	+
Rabins et al., 2007, United States	+	+	+	+
Fillit et al., 2006, United States	--	+	--	--
Scottish Intercollegiate Guidelines Network, 2006, Scotland	+	--	--	+
Total	3 (20.0)	12 (80.0)	8 (53.3)	10 (66.7)

Table 5 provides examples of sex/gender and intersectionality, and dementia-specific person-centred care content extracted from included guidelines and 18 key strategies offered across guidelines for each domain. With respect to sex/gender and intersectionality, most guidelines emphasized the need to accommodate and tailor care to diverse persons living with dementia and caregiver characteristics, referring to: age (i.e., early onset), sexual identity, cultural or Indigenous norms, religious beliefs; learning, physical or sensory disability; low literacy or education, limited financial means, and those in rural or remote locations. Across all guidelines, 6 strategies for doing so emerged; for example, all staff should receive training in person-centred dementia care; tailor assessment, care and support; and involve bilingual or bicultural staff or professional interpreters. Guidelines inconsistently addressed quality of life and some provided limited detail. Across guidelines, 7 strategies emerged for optimizing quality of life; for example, offer a range of activities to promote well-beingg that are tailored to the person’s preferences and stage of dementia; routinely assess activities and interventions to adjust as needed; and offer psychological support for caregivers or family to reduce their care burden and improve quality of life. Few guidelines provided detailed insight on how to support the dignity of person living with dementia, narrowly focusing on identifying abuse or neglect, which is an important issue, rather than addressing how clinicians can respect the dignity of person living with dementia through their own behaviour, and by counseling persons living with dementia and caregivers on how to do so. Across guidelines, 3 strategies emerged: preserve dignity through all stages of dementia, routinely assess and document possible abuse or neglect; and when identified, offer interventions to persons living with dementia and caregivers through medical treatment and referrals. Few guidelines recognized issues of sex or gender in the context of dementia.

**Table 5.** Examples of dementia-specific person-centred care content in included guidelines.

PCC domain	Brief example	Detailed example	Recommended strategies
Sex/gender	Oestrogen is not recommended for the treatment of associated symptoms in women with dementia. (Scottish Intercollegiate Guidelines Network, 2006).	There are more women with dementia, partly because of greater longevity, but also because Alzheimer’s disease is more prevalent among women for reasons that are not known. In addition, because of their greater life expectancy (and tendency to marry men older than themselves), women with dementia are more likely to have an adult child rather than a spouse as caregiver. Unlike an elderly spouse caregiver, who is more likely to be retired, adult child caregivers (most often daughters or daughters-in-law) are more likely to have jobs outside the home and/or to be raising children. These additional caregiver responsibilities may contribute to earlier institutionalization for elderly women with dementia (Abbey et al., 2008).	• Design services to be accessible to all persons living with dementia including those who care for children • Ascertain if women with dementia have sufficient caregiving from family members
Intersectionality	Patient-centred and culturally sensitive care should be provided at all times (Amante et al., 2012).	Care and support providers should provide all staff with training in person centred and outcome-focused care for people living with dementia, which should include respecting the person’s individual identity, sexuality and culture. Service providers should design services to be accessible to as many people living with dementia as possible, including: • People who do not have a carer or whose carer cannot support them on their own • People who do not have access to affordable transport, or find transport difficult to use • People who have other responsibilities (such as work, children or being a carer themselves) • People with learning disabilities, sensory impairment (such as sight or hearing loss) or physical disabilities • People who may be less likely to access health and social care services, such as people from black, Asian and minority ethnic groups (Hort et al., 2010).	• All staff should receive training in person-centred dementia care • Ensure equitable access to dementia related information, support and services • Assessment, care and support should be tailored to: age/early onset, sexual identity, cultural or Indigenous norms, religious beliefs; learning, physical or sensory disability, low literacy or education, limited financial means, and those in rural or remote locations • Assessment for dementia should be based on tools designed for diversity, and on use of multiple tools • Involve bilingual or bicultural staff, or professional interpreters • Refer to ethno-specific support agencies
Dignity	Person-centred care that emphasizes the dignity and autonomy of the patient, should be upheld at all stages irrespective of the patient’s functional status (National Institute for Health and Clinical Excellence, 2018).	Conduct and document an assessment and monitor changes in: living arrangement, safety, care needs, and abuse and/or neglect. With respect to the patient, simple questions such as: “Are you afraid of anyone? Is anyone stealing from you? Has anyone hurt you?” are easy ways to screen for abuse. In addition, the most important care recipient characteristics to look for in assessing for potential abuse are: • Problems with short-term memory; • Psychiatric diagnosis; • Alcohol abuse; • Difficulty interacting with others; • Self-reported conflict with family members and friends; • Feelings of loneliness; and • Inadequate or unreliable support system It is recommended that patients exhibiting three of the seven predictors of potential abuse be targeted for further investigation, although fewer “triggers” also may signal a strong need for preventive measures such as additional support services. Because timely referrals to support services may help mitigate or eliminate circumstances associated with abuse and neglect, thorough assessment and monitoring by the PCP is essential to the safety of both patient and caregiver (Ministry of Health, Malaysia, 2009).	• Preserve safety, comfort and dignity through all stages of dementia • Routinely assess and document possible abuse or neglect • Offer interventions to the patient and caregiver through medical treatment and referrals to community agencies
Quality of life	Careful assessment for any co-morbidities and consideration given to other services that may be required including social services, mental stimulation, occupational therapy, physiotherapy, speech and language therapy. Occupational therapy can benefit patients’ daily functioning and reduce the need for informal care (Toward Optimum Practice, 2017).	Behaviour management may be used to reduce depression in people with dementia. Evidence suggests that reduction of repetitive verbalizations, management of aggression and management of eating behaviours in people with dementia have a positive effect on behaviour and well-being. Multilevel behavioural management interventions may be more effective than individual interventions at improving behaviour and well-being in people with dementia.	• Persons living with dementia must not be under-treated nor forced to receive inappropriate treatment just because they lack decision making capacity or legally appointed guardian • Offer a range of activities to promote well-bring that are tailored to the person’s preferences and stage of dementia • Routinely assess activities and interventions to adjust as needed • Consider sleep management approaches, cognitive rehabilitation or occupational therapy or socialization programs to support functional ability, environmental assessment, and assistive technology • Adjust the environment of the person living with dementia to optimize safety, comfort and functionality • Offer caregivers advice on planning meaningful activities to do with the person they care for • Offer psychological support for caregivers or family to reduce their care burden and quality of life
		For people with dementia, a combination of structured exercise and conversation may help maintain mobility. Recreational activities should be introduced to people with dementia to enhance quality of life and well-being. Individualized activities adapted to maximize the person’s remaining abilities and based on previous interests may be more beneficial to people with dementia than generic activities (Scottish Intercollegiate Guidelines Network, 2006).	

## Discussion

Analysis of the content of 15 guidelines on the overall management of dementia published in 8 countries from 2006 to 2020 revealed inconsistent and limited content across guidelines on person-centred care and support for persons living with dementia or caregivers who are women. With respect to general person-centred care, guidelines most commonly addressed the domains of exchange information, share decisions, enable self-management and address emotions, while few offered content on the domains of manage uncertainty or foster a healing relationship. Regarding dementia-specific person-centred care, most guidelines addressed intersectionality by recognizing the need to accommodate the needs of person living with dementia and caregivers with diverse characteristics and circumstances, but few included content on the domains of quality of life, dignity or sex/gender issues. Even when these general and dementia-specific domains were mentioned, guidance was often brief. Hence, most guidelines included in this analysis offer limited guidance and support to clinicians involved in caring for women affected by dementia.

The findings of this analysis reinforce prior research that revealed little guidance in dementia policies or published research on how to optimize person-centred dementia care for persons living with dementia and caregivers who are women (Bartlett et al., 2018; Chow et al., 2018; Marulappa et al., 2022). This research highlighted a similar paucity of such guidance in dementia guidelines, which are fundamental knowledge resources referred to by clinicians (Shekelle et al., 2012). This study also builds on considerable research that explored barriers of high-quality dementia care. For example, a scoping review of 88 studies published from 1998 to 2015 on implementing evidence-informed dementia care identified numerous barriers that were largely at the organizational (e.g., time constraints, workload, leadership) and clinician (e.g., lack of knowledge and training, resistance to change) levels, and found that education of professionals was the most common strategy employed to improve dementia care (Lourida et al., 2017). Interviews with 129 healthcare managers, clinicians and service staff, and 17 person living with dementia and 31 caregivers also identified barriers of post-diagnostic support for persons living with dementia including lack of clinician time and knowledge, inconsistent follow-up, lack of a holistic approach to care and limited planning for future needs; and recommended support to enable non-specialists to deliver dementia care (Wheatley et al., 2021). The same study generated 20 desired clinician behaviours to improve timely identification and management of needs, understanding of how to manage dementia, provision of support for emotional and psychological wellbeing of persons living with dementia and caregivers, and the integration of other support services (Bamford et al., 2021). Our work is novel because it explored how guidelines do and could overcome the many barriers of high-quality dementia care by providing clinicians with knowledge and strategies for addressing the needs of person living with dementia and caregivers. Unlike prior research that focused on critically appraising the methodological quality of dementia guidelines (Elo & Kyngäs, 2008; Ngo & Holroyd-Leduc, 2015), our analysis specifically examined dementia guidelines for content that would help clinicians deliver person-centred dementia care tailored for persons living with dementia and caregivers who are women. In so doing, it supplements the aforementioned qualitative research (Bamford et al., 2021; Wheatley et al., 2021) by revealing strategies that clinicians can apply to achieve holistic, person-centred dementia care. Our work also expands upon a prior analysis of dementia guidelines that specifically focused on intersectional factors, and like our study, found that most guidelines recognized the need to tailor dementia care to the needs of diverse persons (James et al., 2022).

Several key implications emerge from this study for future policy, practice and research. While no single guideline offered comprehensive guidance to clinicians on how to optimize dementia care, compiling content across guidelines generated considerable guidance (32 general and 18 dementia-specific strategies) to optimize person-centred care. The identified gaps help to improve guidance on dementia care for women in two ways: by revealing how to enhance content on person-centred care, which can help clinicians tailor care for women who vary by a range of intersectional factors; and by revealing the need to specifically acknowledge disparities among women, which can encourage clinicians to apply the aforementioned strategies. These strategies can serve as the basis for developing new or strengthening existing dementia care policies or programs, education and point-of-care tools for clinicians, and measures for evaluating dementia care in the context of quality improvement or future research. Given the long-standing emphasis on person-centred dementia care, yet lack of wide agreement on explicit processes to achieve its broad conceptual components (Fazio et al., 2018), the strategies identified here for achieving person-centred care, both general and dementia-specific, extend prior conceptual frameworks.

This study found that many guidelines lacked person-centred care content, and even when mentioned, details were brief, providing little assistance to clinicians who have advocated for greater guidance (Bourque & Foley, 2020; Koch et al., 2010; Mansfield et al., 2019) given lack of training in person-centred care (Anderson & Gagliardi, 2021a; 2021b). This raises the question of why included dementia guidelines, and guidelines on other topics did not address person-centred care for women (Abuwa et al., 2023; Gagliardi et al., 2019). Notably, few guidelines included in this study involved women living with dementia or caregivers in their development. It is now well-recognized that guidelines better address patient and family perspectives and preferences when those stakeholders are involved in establishing guideline topics or questions, assembling and reviewing guideline content, and formulating the recommendations (Armstrong et al., 2016). Resources are available to assist developers in engaging patients and families in guideline development (Kim et al, 2020; Kim et al, 2021; The Guidelines International Network (GIN) PUBLIC Working Group, 2015). Those resourcesand the strategies identified in this research could be used by developers of dementia guidelines to expand the person-centred content of their guidelines, particularly for domains that were not well addressed: fostering a healing relationship, managing uncertainty, promoting dignity and enhancing quality of life.

Despite the disproportionate impact of dementia on women, the near absence of content related to sex and gender represents a considerable gap in guidance for clinicians on how to enhance person-centred care for women living with dementia and caregivers, which is needed to overcome a lack of training among clinicians in women’s health (Anderson & Gagliardi, 2021a), documented disparities in the quality of dementia care experienced by women (Sourial et al., 2020), little research on how to alleviate the burden on women caregivers, and a lack of research on support for women living with dementia (Bartlett et al., 2018; Erol et al., 2015). Future research is urgently needed to more fully understand the experiences and needs of women living with dementia and caregivers, and how to promote and support person-centred care for women affected by dementia, knowledge needed to enable clinicians and health systems to optimize care and associated outcomes. In the meantime, guidelines could be strengthened by noting the disproportionate impact of dementia on women (Bott et al., 2016; Prince et al., 2015; Sourial et al., 2020); including guidance generated in prior research based on consultations with women on how to improve person-centred care (Filler et al., 2020; Gagliardi et al., 2020), and specific guidance compiled in this research on how to do so; for example, fully assess women’s circumstances to develop an individualized plan and ascertain if women with dementia have sufficient caregiving from family members or other supports.

This study featured multiple strengths. We used rigorous methods to search for and extract data from guidelines, and complied with relevant research reporting criteria (Elo & Kyngäs, 2008; Hsieh & Shannon, 2005; O’Brien et al., 2014). To guide data extraction and analysis, we employed established frameworks of general and dementia-specific person-centred care (Fazio et al., 2018; McCormack et al., 2011). To guide future guideline development, we charted gaps in guideline content, which identified guidelines that better addressed person-centred care and guidelines that should be strengthened in future iterations. To guide future policy and practice, we compiled examples of person-centred content and strategies to achieve person-centred care. We must note a few limitations. Despite employing a comprehensive search strategy, we may not have identified all relevant guidelines. This was further limited by including only guidelines on the overall management of dementia, and those published in English language. However, using this research and its findings as a guide, others can examine the content of guidelines on specific dementia topics and guidelines published in other languages.

## Conclusions

Despite the disproportionate impact of dementia on persons living with dementia and caregivers who are women, and the long-standing emphasis on person-centred dementia care, this analysis of 15 guidelines on the overall management of dementia revealed inconsistent and limited content across guidelines on person-centred care and support for women affected by dementia. Hence, guidelines, which are tools used by clinicians to inform practice, must be strengthened with guidance to help clinicians achieve person-centred dementia care for women living with dementia and caregivers. Our study revealed 32 general and 18 dementia-specific person-centred care strategies. This compiled knowledge could be used by developers to enhance the content of current or future dementia guidelines, and to develop dementia care policies or programs, education and point-of-care tools for clinicians, and measures for evaluating dementia care in the context of quality improvement or future research. Given the lack of content on sex and gender, and a similar lack of existing research on these issues, future research is essential to understand how to promote and support person-centred dementia care for women affected by dementia.

## Supplemental Material

Supplemental Material - Do clinical guidelines support person-centred care for women affected by dementia: A content analysis Supplemental Material for Do clinical guidelines support person-centred care for women affected by dementia: A content analysis by Nevetda Gengeswaran, Alec Brandwood, Natalie N Anderson, Jessica U Ramlakhan and Anna R Gagliardi in Dementia

## Data Availability

All data generated or analysed during this study are included in this published article and its supplementary information files.
