# Supplementary material for: Do clinical guidelines support person-centred care for women affected by dementia: A content analysis
Source: Dementia (London). 2024 Apr 3;23(4):525–49. doi: 10.1177/14713012241244982 (PMC11059842; doi:10.1177/14713012241244982)
Supplement: Supplemental Material - Do clinical guidelines support person-centred care for women affected by dementia: A content analysis [file sj-pdf-1-dem-10.1177_14713012241244982.pdf]

Supplemental File 1. Characteristics of included guidelines

**Guideline**<br>**Author/Year/Country**	**Developer type**	**Development approach**	**Dementia cause**
Recommendations of the 5th Canadian Consensus Conference on the Diagnosis and Treatment of Dementia (CCCDTD5) (Ismail et al., 2020)<br><br>5th Canadian Consensus Conference on the Diagnosis and Treatment of Dementia.<br><br>2020<br><br>Canada	Professional Association	Literature Search<br><br>AGREE II<br><br>GRADE<br><br>Delphi-based process	Type: Alzheimer's Disease, Lewy body Dementia, Parkinson's disease Dementia, Vascular Dementia<br><br>Stage: All
Dementia: Assessment, Management, and Support for people living with dementia and their careers (National Institute for Health and Care Excellence, 2018)<br><br>National Institute for Health and Care Excellence<br><br>2018<br><br>United Kingdom	Professional Association	Meta- analyses<br><br>Thematic syntheses<br><br>Literature reviews<br><br>GRADE<br><br>Social care practitioners involved	Type:<br>Mild Cognitive Impairment, Alzheimer's Disease, Lewy body Dementia, Mixed Dementia, Vascular Dementia<br><br>Stage: All
Cognitive Impairment- Part 2: Diagnosis to Management (Toward Optimum Practice, 2017)<br><br>Toward Optimum Practice Cognitive Impairment CPG Committee, Alberta Medical Association<br><br>2017<br><br>Canada	Professional Association	Literature review<br><br>AGREE II<br><br>ADAPTE<br><br>Target users involved	Type:<br>Alzheimer's Disease, Dementia with Lewy Bodies, Fronto-temporal Lobe Dementia, Vascular Dementia<br><br>Stage: Mild, moderate
Clinical Practice Guidelines and Principals of Care for people with Dementia (Guideline Adaptation Committee, 2016)<br><br>Cognitive Decline Partnership Centre<br><br>2016<br><br>Australia	Professional Association	Systematic Review<br><br>ADAPTE<br><br>GRADE	Type:<br>Alzheimer's Disease, Fronto-temporal Lobe Dementia , Dementia with Lewy Bodies, Parkinson's Disease dementia, Vascular Dementia<br><br>Stage: early, moderate, advanced

Cognitive impairment: Recognition, Diagnosis, and Management in Primary Care (The Government of British Colombia, 2016)<br><br>Government of British Columbia<br><br>2016<br><br>Canada	Government	Systematic review	Type<br>Alzheimer's Disease, Dementia with Lewy bodies, Fronto-temporal Lobe Dementia, Parkinson's Disease dementia, Vascular Dementia<br><br>Stage: all
Ministry of Health Clinical Practice Guidelines: Dementia (Nagaendran et al., 2013)<br><br>Ministry of Health Singapore<br><br>2013<br><br>Singapore	Government	Systematic review<br><br>Patient representatives involved	Type:<br>Mild Cognitive Impairment, Alzheimer's Disease, Fronto-temporal Lobe Dementia , Dementia with Lewy Bodies , Parkinson's Disease dementia, Vascular Dementia<br><br>Stage: All
EFNS Guidelines for the diagnosis and management of Alzheimer's disease (Hort et al., 2010)<br><br>Hort et al.<br><br>2010<br><br>Czech Republic	Professional Association	Systematic review<br><br>EFNS Grade of recommendations and levels of evidence	Type:<br>Alzheimer's Disease<br><br>Stage: All
Clinical Practice Guideline on the Comprehensive Care of People with Alzheimer's Disease and other Dementias (Ministry of Health Social Services and Equality, 2010)<br><br>Ministry of Health, Social Services, and Equality Catalunya<br><br>2010<br><br>Spain	Government	Systematic review<br><br>SIGN Grade of recommendations and levels of evidence<br><br>Involved caregiver representatives<br><br>Involved dementia-care organizations	Type:<br>Mild Cognitive Impairment, Alzheimer's Disease, Fronto-temporal Lobe Dementia , Dementia with Lewy Bodies , Parkinson's Disease dementia, Vascular Dementia<br><br>Stage: All
Clinical Practice Guidelines for the Management of Dementia (Ministry of health, Malaysia, 2009)<br><br>Ministry of Health Malaysia	Government	Systematic review<br><br>AGREE	Type:<br>Mild Cognitive Impairment, Alzheimer's Disease, Fronto-temporal Lobe Dementia , Dementia with Lewy Bodies ,

2009<br><br>Malaysia		SIGN Grade of recommendations and levels of evidence<br><br>Involved caregiver representatives<br><br>Involved stakeholders	Mixed dementia, Vascular Dementia<br><br>Stage: All
Group Health Dementia and Cognitive Impairment Diagnosis and Treatment Guideline (Amante et al., 2012)<br><br>Group Health<br><br>2012<br><br>United States	Professional Association	Systematic review	Type:<br>Mild Cognitive Impairment, Alzheimer's Disease, Dementia with Lewy bodies , Vascular Dementia<br><br>Stage: All
Clinical Practice Guidelines and Care Pathways for People with Dementia Living in the Community (Abbey et al., 2008)<br><br>Abbey et al.<br><br>2008<br><br>Australia	Professional Association (University)	Review and synthesis of existing guidelines<br><br>AGREE<br><br>NHMRC rating scale<br><br>In consultation with stakeholders and consumers	Type:<br>Mild Cognitive Impairment, Alzheimer's Disease, Fronto-temporal Lobe Dementia , Dementia with Lewy Bodies, Vascular Dementia<br><br>Stage: All
Guideline for Alzheimer's Disease Management: Final Report (California Workgroup on Guidelines for Alzheimer's Disease Management, 2008)<br><br>California Workgroup on Guidelines for Alzheimer's Disease Management<br><br>2008<br><br>United States	Government	Systematic review<br><br>Involved consumer representatives	Type:<br>Alzheimer's Disease<br><br>Stage: All
The Practice Guideline for the Treatment of Patients with Alzheimer's Disease and Other Dementias (Second Edition) (Rabins et al., 2007)	Professional Association	Systematic review	Type:<br>Alzheimer's Disease , Dementia with Lewy bodies, Fronto-temporal lobe dementia, Parkinson's disease

American Psychiatric Association<br><br>2007<br><br>United States			dementia, Vascular Dementia<br><br>Stage: All
Recommendations for Best Practices in the Treatment of Alzheimer's Disease in Managed Care (Fillit et al., 2006)<br><br>Fillit et al.<br><br>2006<br><br>United States	Professional Association	Systematic review	Type:<br>Alzheimer's Disease<br><br>Stage: All
Management of patients with dementia. A national clinical guideline (Scottish Intercollegiate Guidelines Network, 2006)<br><br>Scottish Intercollegiate Guidelines Network,<br><br>2006<br><br>Scotland	Professional Association	Systematic review<br><br>SIGN Grade of recommendations and levels of evidence<br><br>Involved patient representatives	Type:<br>Alzheimer's Disease , Dementia with Lewy bodies, Fronto-temporal lobe dementia, Vascular Dementia<br><br>Stage: All

Additional File 2. Data extracted on general aspects of patient-centred care

Guideline	Foster the Relationship	Exchange Information	Address emotions	Manage Uncertainties	Share Decisions	Enable self-management
Canada 2020 (Ismail et al., 2020)	--	Primary care health professionals should be vigilant for potential symptoms of cognitive disorders in older or at-risk individuals, including but not limited to: reported cognitive symptoms by the patient or an informant.<br><br>In persons at elevated risk for cognitive disorders (such as very advanced age, pre-existing brain diseases such as Parkinson's disease, a recent episode of delirium, or risk factors such as diabetes) it is reasonable to ask the patient (and an informant, if	--	Given that the presence of brain amyloid and/or tau in cognitively normal people is of uncertain significance, we discourage the use of amyloid and tau imaging without memory decline, outside of the research setting. The medical community should be clear in its discussion with patients, the media, and the general population that the presence of brain	Decisions related to deprescribing of cognitive enhancers should take into consideration the patient's preferences (for individuals who are capable of making treatment decisions), their prior expressed wishes (if these are known), and in collaboration with family or substitute decision makers for individuals who are incapable of	Caregiver burden is a major determinant of hospitalization and nursing home placement. It should be regularly assessed in the follow-up of patients with dementia. This can be done with structured scales such as the Zarit Burden Interview,62 etc.<br><br>Psychoeducational interventions for caregivers aim at the development of problem-focused coping strategies while

		available) about concerns regarding memory.<br><br>The distinction between MCI and dementia is important and is currently made on the basis of clinical assessment of cognition and function. For screening purposes, examining the complaint with the patient and a family member and proceeding with an objective assessment of cognition and functional impairment should be done.<br><br>To obtain information in addition to that provided by the other psychometric screening tools, or if the patient is unable to answer the questions on the screening tools (lack of time or uncooperative), having the caregiver complete a questionnaire for identifying a cognitive and/or functional change, such as the Ascertain Dementia 8 (AD-8) questionnaire or the Informant Questionnaire on cognitive decline in the elderly (IQCODE) 33 is recommended.<br><br>Rapid screening of functional autonomy should be completed by an objective assessment with the patient and a family member using the Pfeffer Functional Activities Questionnaire (FAQ)34 or the Disability Assessment for Dementia (DAD).<br>If a personality, behavior, or		amyloid and/or tau in normal people is of unclear significance at the present time.<br><br>It is reasonable to ask the patient and an informant about concerns regarding cognition and behavior.<br><br>For patients with a negative corroborative history, reassurance should be provided, and follow-up offered if the patient or informant sources note deterioration in the future in any of the domains of cognition, function, or behavior.	providing informed consent.	psychosocial interventions address the development of emotion-focused coping strategies. These can include education, counseling, information regarding services, enhancing carer skills to provide care, problem solving, and strategy development. We recommend considering psychosocial and psychoeducational interventions for caregivers of people living with dementia.

		mood change has been observed, an objective assessment of the behavioral and psychological symptoms of dementia (BPSD) with the patient and a family member using the short version of the Neuropsychiatric Inventory (NPI-Q),36 Mild Behavioural Impairment Checklist (MBI-C)37 or if a mood change has been observed with the Patient Health Questionnaire-9 (PHQ).38 1A (93%)<br><br>What important information can be gained from an informant, using which measures?<br>1. Due to variability in insight into cognitive, functional, and behavioral changes, report from a reliable informant is an essential component for the assessment of patients with suspected neurocognitive disorders at all settings.<br><br>2. The use of standardized tools to obtain informant report on changes in cognition, function, and behavior increases the diagnostic accuracy when combined with patient-related measures and therefore is recommended.<br><br>3. We recommend using one or more informant-based tools that cover cognitive, functional, and behavioral aspects. Specific tools can be selected based on the				

		need for comprehensive assessment versus efficiency depending upon the setting<br><br>4. There is ongoing development of informant-based tools, and based on the current evidence we recommend tools that: measure informant's report of cognitive changes (eg, ECog)39; measure informant's report on cognitive and functional changes (eg, AD8, IQCODE, Quick Dementia Rating System [QDRS]40); measure informant's report on functional changes combined with cognitive assessment as an alternative (eg, FAQ, Lawton-Brody IADL,41 4-item IADL scale [4-IADL],42 Amsterdam IADL questionnaire [A-IADL-Q]43); measure informant's report on behavioral changes (eg, NPI-Q, MBI-C).<br><br>Obtaining corroborative history is essential, and has prognostic significance. Reliable informant information should be obtained for changes in cognition, function, and behavior/neuropsychiatric symptoms (ie, new onset symptoms vs chronic or longstanding symptoms).<br><br>Tracking response to treatment and change over time should be individualized, and requires a multi-dimensional approach. It				

		should not rely on a single tool or clinical domain and requires caregiver or reliable informant input.<br><br>Global assessment can be done with validated and simple tools that integrate input from the caregiver such as the Informant Questionnaire on Cognitive Decline in the Elderly (IQCODE), the HABC-Monitor, 61 etc.				
United Kingdom 2018 (National Institute for Health and Care Excellence , 2018)	--	Involving people in decision-making<br>If needed, use additional or modified ways of communicating (for example visual aids or simplified text).<br><br>Consider using a structured tool to assess the likes and dislikes, routines and personal history of a person living with dementia.<br><br>Providing information<br>Provide people living with dementia and their family members or carers (as appropriate) with information that is relevant to their circumstances and the stage of their condition.<br><br>At diagnosis, offer the person and their family members or carers (as appropriate) oral and written information that explains:<br>• what their dementia subtype is and the changes to expect as the condition progresses	When people living with dementia or their carers have a primary care appointment, assess for any emerging dementia-related needs and ask them if they need any more support.<br><br>For people living with dementia who experience agitation or aggression, offer personalized activities to promote engagement, pleasure and interest.		It is not mandatory to apply the recommendations, and the guideline does not override the responsibility to make decisions appropriate to the circumstances of the individual, in consultation with them and their families and carers or guardian.<br>This guideline addresses how dementia should be assessed and diagnosed. It covers person- centred care and support, tailored to the specific needs of each person living with dementia. As part of this, it can help professionals involve people living with dementia and their carers in decision-making, so they can get the care and support they need.	Offer carers of people living with dementia a psychoeducation and skills training intervention that includes:<br>• developing personalized strategies and building carer skills<br>• training to help them provide care, including how to understand and respond to changes in behaviour<br>• training to help them adapt their communication styles to improve interactions with the person living with dementia<br>• advice on how to look after their own physical and mental health, and their emotional and spiritual wellbeing<br>• information about relevant services (including support services and psychological therapies for carers) and how to access them

		• which healthcare professionals and social care teams will be involved in their care and how to contact them<br>• if appropriate, how dementia affects driving, and that they need to tell the Driver and Vehicle Licensing Agency (DVLA) and their car insurer about their dementia diagnosis<br>• their legal rights and responsibilities<br>• their right to reasonable adjustments (in line with the Equality Act 2010) if they are working or looking for work<br>• how the following groups can help and how to contact them:<br>• local support groups, online forums and national charities<br>• financial and legal advice services<br>• advocacy services.<br><br>If it has not been documented earlier, ask the person at diagnosis:<br>• for their consent for services to share information<br>• which people they would like services to share information with (for example family members or carers)<br>• what information they would like services to share. Document these decisions in the person's records.<br><br>After diagnosis, direct people and their family members or carers			For all recommendations, NICE expects that there is discussion with the patient about the risks and benefits of the interventions, and their values and preferences. This discussion aims to help them to reach a fully informed decision.<br>Interventions that could be used. We use ‘consider’ when we are confident that an intervention will do more good than harm for most patients, and be cost effective, but other options may be similarly cost effective. The choice of intervention, and whether or not to have the intervention at all, is more likely to depend on the patient’s values and preferences than for a strong recommendation, and so the healthcare professional should spend more time considering and discussing the options with the patient.<br>People have the right to be involved in discussions and make informed decisions about their care	

		(as appropriate) to relevant services for information and support<br><br>For people who do not want follow-up appointments and who are not using other services, ask if they would like to be contacted again at a specified future date.<br><br>Ensure that people living with dementia and their carers know how to get more information and who from if their needs change.<br><br>Tell people living with dementia (at all stages of the condition) about research studies they could participate in.<br><br>At the initial assessment take a history (including cognitive, behavioural and psychological symptoms, and the impact symptoms have on their daily life). If possible, from someone who knows the person well (such as a family member).<br><br>When taking a history from someone who knows the person with suspected dementia, consider supplementing this with a structured instrument such as the Informant Questionnaire on Cognitive Decline in the Elderly (IQCODE) or the Functional Activities Questionnaire (FAQ).<br><br>Care coordination			Encourage and enable people living with dementia to give their own views and opinions about their care.<br>Offer early and ongoing opportunities for people living with dementia and people involved in their care to discuss:<br>• the benefits of planning ahead<br>• lasting power of attorney (for health and welfare decisions and property and financial affairs decisions)<br>• an advance statement about their wishes, preferences, beliefs and values regarding their future care<br>• advance decisions to refuse treatment<br>• their preferences for place of care and place of death.<br>Explain that they will be given chances to review and change any advance statements and decisions they have made.<br><br>At each care review, offer people the chance to review and change any advance statements and decisions they have made.	

		Named professionals should:<br>• arrange an initial assessment of the person's needs, which should be face to face if possible<br>• provide information about available services and how to access them<br>• ensure that people are aware of their rights to and the availability of local advocacy services, and if appropriate to the immediate situation an independent mental capacity advocate<br><br>Before starting antipsychotics, discuss the benefits and harms with the person and their family members or carers (as appropriate). Consider using a decision aid to support this discussion.<br><br>Stop treatment with antipsychotics:<br>• if the person is not getting a clear ongoing benefit from taking them and<br>• after discussion with the person taking them and their family members or carers (as appropriate).<br><br>Care providers should provide additional face-to-face training and mentoring to staff who deliver care and support to people living with dementia. This should include how to monitor and respond to the lived			Advance care planning: Involve the person and their family members or carers (as appropriate) as far as possible, and use the principles of best-interest decision-making if the person does not have capacity to make decisions about their care.	

		experience of people living with dementia, including adapting communication styles<br><br>Consider giving carers and/or family members the opportunity to attend and take part in staff dementia training sessions				
Canada 2017 (Toward Optimum Practice, 2017)	--	--	Enlist support from family, friends and community resources (e.g., home care, day programs, respite) to maximize functioning and ease caregiver burden	--	Actively encourage patients (while they retain capacity) to update their will and complete both a personal directive and an enduring power of attorney.<br><br>DISCONTINUATION OF CHOLINESTERASE INHIBITORS. The benefits of discontinuing cholinesterase inhibitors should be considered and balanced against possible worsening of cognition and function after stopping the agent in an individual who has been taking the agent for a prolonged period of time. It is suggested that cholinesterase inhibitors be discontinued when: Patient/caregiver/decision-maker decides to stop the agent after being informed of the risks and benefits of continuing and discontinuing the cholinesterase inhibitors	GENERAL MANAGEMENT CHECKLIST.<br>• Determine how medications are being managed. If there is evidence of medication nonadherence, suggest using reminder aids and/or request that a caregiver manage medications.<br>• Remain vigilant for and strive to mitigate safety risks particularly in relation to driving, cooking, wandering, financial management and abuse while supporting functional independence wherever possible.<br><br>BEHAVIOURAL AND PSYCHOLOGICAL SYMPTOMS OF DEMENTIA<br>• Advise caregivers to re-direct and distract the patient, and remove triggers when possible to prevent or ease agitation and aggression.<br>• Recommend specific training for caregivers so they can provide person-

						centred care, manage behaviours, and learn communication skills.<br>• Consider tracking devices, motion detection devices, and home alarms to assist with detecting wandering and locating lost patients.
Australia 2016 (Guideline Adaptation Committee, 2016)	--	Health and aged care professionals should talk to the person with dementia and their carer(s) and family about the symptoms of dementia, treatments and services. Written information (such as brochures) should also be provided.<br><br>Health and aged care professionals should discuss with the person any need for information to be shared. However, as the condition progresses and the person with dementia becomes more dependent on family or other carers, decisions about sharing information (with other health professionals or substitute decision makers) should be made in the context of the person's capacity to make decisions. If information is to be shared, this should be done only if it is in the best interests of the person with dementia.<br><br>A diagnosis of dementia should be made only after a comprehensive assessment, which should include:	As the transition to residential care is a particularly difficult step for the person living with dementia, their family and community, health and aged care professionals should display sensitivity and could consider organizing support from the community and Indigenous staff members at this time.<br><br>Medical practitioners should be aware that people with a history of depression and/or self-harm may be at	--	Health and aged care professionals should discuss with the person with dementia, while he or she still has capacity, and his or her carer(s) and family the use of:<br>• an Enduring Power of Attorney and enduring guardianship<br>• Advance Care Plans. Advance Care Plans should be revisited with the person with dementia and his or her carer(s) and family on a regular basis and following any significant change in health condition or circumstance. Advance Care Plans should be completed or updated at the time of assessment undertaken by the Aged Care Assessment Team. The medical practitioner should recognize that people have the right to know their diagnosis and the right not to know their diagnosis. In rare	Health and aged care professionals should inform the person with dementia, their carer(s) and family about advocacy services and voluntary support, and should encourage their use. If required, such services should be available for both the person with dementia and their carer(s) and family independently of each other.<br><br>Carer(s) and family should be supported to care for the person with dementia. They should be offered education and training to enable them to develop skills in managing the symptoms of dementia and be offered respite when needed. Carer(s) and family should be given information about coping strategies to maintain their own wellbeing and be supported to maintain their overall health and fitness.<br><br>The medical practitioner

		• history taking from the person<br>• history taking from a person who knows the person well, if possible<br><br>The diagnosis of dementia should be communicated to the person with dementia by a medical practitioner.<br><br>Conflicts, such as when the carer(s) and family request the diagnosis not be communicated to the person with dementia should be resolved by further discussions over time if necessary.<br><br>The medical practitioner should provide information about dementia in a clear manner and emphasize that progression is often slow, symptomatic treatments are available and that research is striving to find cures, though so far without success.<br><br>Following a diagnosis of dementia, health and aged care professionals should, unless the person with dementia clearly indicates to the contrary, provide them and their carer(s) and family with written and verbal information in an accessible format about:<br>• the signs and symptoms of dementia<br>• the course and prognosis of the condition	particular risk of depression, self-harm or suicide following a diagnosis of dementia, particularly in the first few months post diagnosis. While such reactions are believed to be uncommon, counselling should be offered as an additional way to support the person during this time.<br><br>Health and aged care professionals should be aware that people with dementia, their carer(s) and family members may need ongoing support to cope with the difficulties presented by the diagnosis.		cases where the person with dementia indicates that they do not wish to be told his or her diagnosis, this wish should be respected.<br>Health and aged care managers should coordinate and integrate, referral, transitions and communication across all agencies involved in the assessment, treatment, support and care of people with dementia and their carer(s) and families, including jointly agreeing on written policies and procedures. People with dementia and their carers and families should be involved in planning local policies and procedures.<br>Care coordinators should ensure that care plans are developed in partnership with the person and his or her carer(s) and family and based on a comprehensive assessment including the person with dementia's life history, social and family circumstance, and goals and preferences, as well as the person’s	should ensure that carer(s) and family are supported to manage this situation and that the consequences of this decision are managed (e.g., driving).<br><br>People with a diagnosis of dementia, particularly those living alone, should be provided with information about how to join a social support group.<br><br>In most cases, training the carer(s) and family to provide care, forming specific strategies to address behaviours that are most upsetting, making small changes to the living environment and finding interesting and enjoyable activities for the person to do should be tried first.<br><br>The person with dementia should be encouraged to exercise, eat well, keep doing as much for themselves as possible and stay socially connected in their local community. Staff and carer(s) should be taught how to encourage independence.<br><br>Support programs should be tailored to the needs of the individual and delivered

		• treatments<br>• sources of financial and legal advice, and advocacy<br><br>Health and aged care professionals should ensure that the person with dementia and his or her carer(s) and family are provided with written and verbal information regarding appropriate services available in the community (including those offered by Alzheimer's Australia, Carers Australia, Aged Care Assessment Teams and My Aged Care). Any advice and information given should be recorded.<br><br>People with dementia who develop behavioural and psychological symptoms should be offered a comprehensive assessment at an early opportunity by a professional skilled in symptom assessment and management. This should involve their carer(s) and families as appropriate and include;<br>• analysis of the behaviours (e.g., antecedent [triggers], behaviour description and consequence [ABC approach]), frequency, timing and presentation<br>• assessment of the person with dementia's physical and mental health<br>• their level of pain or discomfort<br>• whether they are experiencing	Carers are at an increased risk of poor health and their needs should be assessed and reviewed regularly by their own health practitioner. Carer and family needs should be addressed regularly, including if the person with dementia has entered residential care, and after their death.<br><br>The person with dementia, their carer(s) and family should be offered respite appropriate to their needs. This may include in-home respite, day respite, planned activity groups and residential respite.		physical and mental health needs, routines and current level of functioning and abilities. Care coordinators should ensure the coordinated delivery of health and aged care services for people with dementia. This should involve:<br>• a care plan developed in partnership with the person and his or her carer(s) and family that takes into account the changing needs of the person<br>• assignment of named health and/or aged care staff to operate the care plan<br>• formal reviews of the care plan at a frequency agreed between professionals involved and the person with dementia and/or their carer(s) and family.<br>Health and aged care staff should be trained to develop individual care plans (in partnership with the person with dementia's carer(s) and family) that provide a clear crises plan to anticipate severe behavioural and psychological symptoms of dementia and how to	in the home or at another accessible location. Programs should be delivered over multiple sessions and include:<br>• information regarding relevant services including respite<br>• support and information regarding coping strategies to maintain their own wellbeing including stress management<br><br>Carers and families of people with dementia should be supported to build resilience and maintain overall health and fitness. Where necessary, they should be offered psychological therapy, conducted by a specialist practitioner.<br><br>Health and aged care professionals should provide carers and families with information regarding how to join a mutual support group.<br><br>Following a diagnosis of dementia, health and aged care professionals should, unless the person with dementia clearly indicates to the contrary, provide them and their carer(s) and family with written and

		side effects of medication<br>• physical environmental and interpersonal factors<br>• an assessment of carer(s) health and communication style when interacting with the person with dementia should also be undertaken<br>• understanding the behaviour as a form of communication.<br><br>People with dementia who have received involuntary sedation should be offered the opportunity, along with their carer(s) and family, to discuss their experiences and be provided with a clear explanation of the decision to use urgent sedation. This should be documented in their notes.<br><br>Carer(s) and family should have access to programs designed to provide support and optimize their ability to provide care for the person with dementia. Programs should be tailored to the needs of the individual and delivered in the home or at another accessible location. Programs should be delivered over multiple sessions and include:<br>education regarding dementia and its consequences<br><br>Advance Care Plans must be recorded in the medical notes and care plans and time should	Carer(s) and family should have access to programs designed to provide support and optimize their ability to provide care for the person with dementia.<br><br>Consideration should be given to involving the person with dementia, as well as their carer(s) and family, in support programs.<br><br>Assist people to maintain confidence and a positive self-esteem.		manage violence, aggression and extreme agitation, including de-escalation techniques.<br><br>Carers and families should be respected, listened to and included in the planning, decision making and care and management of people with dementia.<br>In the absence of a valid and applicable advance directive to refuse resuscitation, the decision to resuscitate should take account of any expressed wishes or beliefs of the person with dementia, together with the views of the carer(s) and family and the multidisciplinary team.<br>The views of the person with dementia should always be sought, even when someone else is making decisions on their behalf.<br>Health and aged care managers should coordinate and integrate, referral, transitions and communication across all agencies involved in the assessment, treatment, support and	verbal information in an accessible format about: medico-legal issues, including driving.<br><br>Health and aged care staff should aim to promote and maintain functional and social independence of people with dementia in community and residential care settings. Interventions should address activities of daily living that maximize independence, function and engagement. Intervention should include:<br>• consistency of care staff<br>• stability in living environment<br>• flexibility to accommodate fluctuating abilities<br>• support for people with dementia and their carer(s) and families to participate in tailored activities that are meaningful and enjoyable<br>• assessment and intervention, involving the carer(s) and family wherever possible, to promote independent self-care skills and prevent excess disability, in particular supporting the person with dementia to retain continence<br><br>To assist the carer(s) and

		be taken to discuss these issues with the carer(s), family and support networks.<br><br>Principles of Dignity in Care Engage with family members and carers as care partners.<br><br>Health and aged care professionals should discuss with the person any need for information to be shared. However, as the condition progresses and the person with dementia becomes more dependent on family or other carers, decisions about sharing information (with other health professionals or substitute decision makers) should be made in the context of the person's capacity to make decisions. If information is to be shared, this should be done only if it is in the best interests of the person with dementia.<br><br>Although a very small number of people may choose not to know the diagnosis, it is clear that the majority of people want to be informed and therefore, it is important that health professionals are honest and truthful when communicating the diagnosis to the person with dementia and those close to them. The diagnosis of dementia is never provided without earlier discussion about memory and			care of people with dementia and their carer(s) and families, including jointly agreeing on written policies and procedures. People with dementia and their carers and families should be involved in planning local policies and procedures.<br>Care coordinators should ensure that care plans are developed in partnership with the person and his or her carer(s) and family and based on a comprehensive assessment including the person with dementia's life history, social and family circumstance, and goals and preferences, as well as the person's physical and mental health needs, routines and current level of functioning and abilities.	family help the person with dementia who is experiencing behavioural and psychological symptoms of dementia, carer(s) and family should be offered interventions which involve:<br>• carer skills training in managing symptoms and communicating effectively with the person with dementia<br>• meaningful activity planning<br>• environmental redesign and modification to improve safety and enjoyment<br>• problem solving and management planning<br><br>Carer(s) and family should have access to programs designed to provide support and optimize their ability to provide care for the person with dementia. Programs should be tailored to the needs of the individual and delivered in the home or at another accessible location. Programs should be delivered over multiple sessions and include:<br>• referral to support organizations such as Alzheimer's Australia or Carers Australia<br>• development of

		thinking difficulties. Medical practitioners should discuss the possibility of dementia as a diagnosis during the process of assessment, which may take three to six months to achieve. Discussion of the diagnosis and its consequences may occur gradually over several visits to the medical practitioner, but should occur as early as practicable.<br><br>Following a diagnosis of dementia, health and aged care professionals should, unless the person with dementia clearly indicates to the contrary, provide them and their carer(s) and family with written and verbal information in an accessible format about:<br>• the signs and symptoms of dementia<br>• the course and prognosis of the condition<br>• treatments<br>• sources of financial and legal advice, and advocacy<br><br>Health and aged care professionals should ensure that the person with dementia and his or her carer(s) and family are provided with written and verbal information regarding appropriate services available in the community (including those offered by Alzheimer's Australia, Carers Australia, Aged Care				individualized strategies and building carer skills to overcome specific problems experienced by the person with dementia as reported by the care<br>• training in providing care and communicating most effectively with the person with dementia<br>• training in the use of pleasant and meaningful activities as a strategy to engage the person with dementia<br><br>Health and aged care professionals should inform the person with dementia, their carer(s) and family about advocacy services and voluntary support, and should encourage their use. If required, such services should be available for both the person with dementia and their carer(s) and family independently of each other.<br><br>Following a diagnosis of dementia, health and aged care professionals should, unless the person with dementia clearly indicates to the contrary, provide them and their carer(s) and family with written and verbal information in an accessible format about:

		Assessment Teams and My Aged Care). Any advice and information given should be recorded.				medico-legal issues, including driving.<br><br>Health and aged care staff should aim to promote and maintain functional and social independence of people with dementia in community and residential care settings. Interventions should address activities of daily living that maximize independence, function and engagement. Intervention should include:<br>• consistency of care staff<br>• stability in living environment<br>• flexibility to accommodate fluctuating abilities<br>• support for people with dementia and their carer(s) and families to participate in tailored activities that are meaningful and enjoyable<br>• assessment and intervention, involving the carer(s) and family wherever possible, to promote independent self-care skills and prevent excess disability, in particular supporting the person with dementia to retain continence<br><br>To assist the carer(s) and family help the person with dementia who is

						experiencing behavioural and psychological symptoms of dementia, carer(s) and family should be offered interventions which involve:<br>• carer skills training in managing symptoms and communicating effectively with the person with dementia<br>• meaningful activity planning<br>• environmental redesign and modification to improve safety and enjoyment<br>• problem solving and management planning<br><br>Carer(s) and family should have access to programs designed to provide support and optimize their ability to provide care for the person with dementia. Programs should be tailored to the needs of the individual and delivered in the home or at another accessible location. Programs should be delivered over multiple sessions and include:<br>• referral to support organizations such as Alzheimer's Australia or Carers Australia<br>• development of individualized strategies and building carer skills to

						overcome specific problems experienced by the person with dementia as reported by the care<br>• training in providing care and communicating most effectively with the person with dementia<br>• training in the use of pleasant and meaningful activities as a strategy to engage the person with dementia
Canada 2016 (The Government of British Colombia, 2016)	--	Always involve the caregiver and plan on several visits to establish and inform patient/caregiver of diagnosis.<br><br>Encourage patient to allow collateral information be obtained from family and caregivers to assist with diagnosis. Consider administering the Alzheimer's Questionnaire to a family member or other reliable informant.<br><br>Discussing the possibility of progression to dementia may facilitate patient participation in monitoring their cognitive decline.<br><br>Schedule regular follow-up visits (e.g., every six months) to monitor possible progression of cognitive deficit.<br><br>Pharmacological Management.	Carefully consider mild cognitive impairment diagnosis disclosure to avoid prompting needless anxiety.	--	Discuss advance care planning as early as possible (e.g., refer to Advance Care Planning Guide for aid in discussing sensitive topics like tube feeding. See also No Cardiopulmonary Resuscitation form); and Encourage patient to have an up-to-date will, a power of attorney agreement for financial management, a representation agreement for health management and/or an advance directive.	Consider the following general care and supplementary supports for patients:<br>Mental Health and Specialty Services<br>• Involve mental health teams and resources, such as Community Mental Health Services, to help in distinguishing depression from dementia, and assessing and treating significant behavioural problems and managing caregiver stress<br>Caregiver Support<br>• Discuss needs, coping strategies, support system and stress management with caregiver (respite care through Home & Community Care); and<br>• Aid in co-ordination, communication and planning during transitions

		However, patients and their caregivers should be advised that benefits are limited, and that side effects and drug interactions are common. End points for discontinuation of medication should be discussed.<br><br>Initiation of therapy. Decision to initiate AChEI therapy requires an individualized patient assessment, involving the patient and caregivers in the following discussion points:<br>• Clinician, patient, and caregiver expectations of benefit with AChEI therapy.<br>• Presence of comorbidities and life expectancy.<br>• Potential drug interactions with concurrent medications.<br>• Ability of the patient or caregiver to adhere to pharmacotherapy.<br>• Potential benefits as compared to potential harms of AChEI therapy.<br>• Patient and caregiver preferences, including cost of therapy.<br><br>If a trial of AChEI therapy is to be initiated, develop and implement a monitoring plan:<br>Encourage caregivers to maintain a written record of symptoms, adverse drug reactions, sleep disturbances, and personal impressions to support ongoing patient assessment.				between care environments.<br><br>Consider the following general care and supplementary supports for patients:<br>a. Memory<br>• Aids like calendars, diaries and telephone reminders;<br>• Keeping keys, glasses, wallet in same designated place (“landing spot”); and<br>• Accompaniment to appointments<br>c. Nutrition<br>• Weigh regularly to monitor for weight loss;<br>• Have the caregiver monitor the refrigerator for food safety; and<br>• Meal support services (e.g., delivered prepared meals or pre-prepared frozen foods).<br>d. Shopping<br>• Shopping assistance from caregiver<br>f. Medication Management<br>• Use blister packages/dossette trays and suggest caregiver supervision to improve safety and compliance<br>j. Driving<br>• To supplement or replace driving encourage patient to register with HandyDart and TaxiSavers.

Singapore 2013 (Nagaendran et al., 2013)	--	Patients' and, where appropriate, their family's preferences for disclosure should be sought with respect to the diagnosis of dementia and acted upon accordingly<br><br>Pharmacotherapy should not be used in isolation in the management of dementia but in conjunction with nonpharmacological management including education and counselling of patient and caregiver<br><br>The current evidence for the use of cognitive enhancers is generally based on clinical trials of up to 1 year duration. The use of cognitive enhancers for longer periods will need to include a detailed discussion with the patient and caregivers on the overall benefit of treatment and specific needs of the patient<br><br>Potential side-effects and risk/benefit ratio of antipsychotic medication should be discussed with patients and/or caregivers<br><br>In general, the diagnosis of dementia should be disclosed to the patient, unless explicitly stated otherwise<br><br>The doctor should also be mindful of the impact the diagnosis can have on the	The complications of dementia can be broadly divided into behavioural and psychological symptoms, functional problems and social problems. These should be evaluated in all patients with dementia as these issues are the major causes of stress on the caregiver and assessment would enable the clinician to target subsequent management effectively.<br><br>Where appropriate, respite care may be offered to relieve the burden of caregiving on caregivers of persons with dementia.	--	The decision to start antipsychotic therapy to control behavioural problems in dementia patients should be made in consultation with the patient and family, after careful consideration of the benefit, adverse-effects and co-morbidities<br>Persons with dementia deemed to have decision making capacity (after clinical evaluation) are encouraged to make a Lasting Power of Attorney (LPA)<br>Advance care planning with regard to cardiopulmonary resuscitation (CPR) should be encouraged given the poor outcomes of CPR in advanced dementia<br>If the clinician is not inclined to perform a brain scan, there is immense value in discussing the matter with the caregivers and in securing their agreement not to order a neuroimaging procedure.<br>A trial of withdrawal of symptomatic treatment should be considered when the harm	Pharmacotherapy should be part of a multi-pronged strategy to dementia management that encompasses a well-established diagnosis and consideration of stage-specific challenges, such as education of patient and caregiver, nonpharmacological measures and comprehensive caregiver psychosocial intervention<br><br>A Multi-component and individualized caregiver interventions should be considered for holistic dementia care.<br><br>Pharmacotherapy should be part of a multi-pronged strategy to dementia management that encompasses a well-established diagnosis and consideration of stage-specific challenges, such as education of patient and caregiver, nonpharmacological measures and comprehensive caregiver psychosocial intervention.<br><br>In the mild stage, the focus is on maintenance of patient independence and autonomy, whereas in the

		patient's life and family relationships. The communication should therefore be conducted sensitively and empathically, and should include a discussion on treatment options and available support services both in the hospital and the community.<br><br>Caregivers of patients with young onset dementia should receive adequate counselling on the disease process, community resources and financial assistance<br><br>Caregivers of persons with advanced dementia should be provided with adequate information on available community and medical resources<br><br>Patients' and, where appropriate, their family's preferences for disclosure should be sought with respect to the diagnosis of dementia and acted upon accordingly<br><br>Although patients generally would like to know the truth about their own medical condition, the rights of those who do not want to know should also be respected. Health care professionals should therefore seek to understand their patients' preferences with respect to the diagnosis of	In disclosure, the doctor should also be mindful of the impact the diagnosis can have on the patient's life and family relationships.<br><br>Identification of caregiver's stress can allow targeted family education and counselling at tertiary dementia care centres, or even dementia day-care centres, and this has been shown to reduce institutionalizati on.<br><br>Interventions for family caregivers are important for the following reasons:<br>3) Caregivers who face much negative consequences as a result of		outweighs the benefit, and should be undertaken only after careful discussion with the patient and caregiver.<br>Respect for autonomous decision making is a fundamental ethical and legal right of a mentally competent individual. This right of self-determination should be respected to the fullest possible extent, even in dementia or conditions associated with cognitive impairment.<br>In deciding if a patient with dementia possesses adequate capacity with respect to making a particular decision, a clinical evaluation of the following functional abilities should be made:<br>a. Ability to express a choice<br>b. Ability to understand information provided<br>c. Ability to appreciate significance of information and relevance to self<br>d. Ability to manipulate information rationally before arriving at a decision	advanced stages, care and psychosocial issues predominate. Thus, a comprehensive multi-pronged strategy to dementia management involves skillful adjustment of pharmacotherapy depending on the stage of disease, done in conjunction with caregiver education, non-pharmacological measures and comprehensive caregiver psychosocial intervention.<br><br>Caregiver intervention can take several forms and they include:<br>2) Home based intervention<br>4) Technology-based interventions<br>6) Psychoeducation and skills training for family caregivers, professional caregivers and domestic helper<br>Given the rapid advancement and easy availability of technology, it should be harnessed more fully to make help available to more family caregivers in a continuous, timely and economical way.<br><br>For patients who are assessed to be unsafe for

		dementia and act appropriately according to their choice<br><br>When informing the diagnosis, the doctor needs to take into account the patient and family's prior knowledge and their perception of the problems<br><br>They should be given ample opportunity to ask questions and seek clarification from the doctor<br><br>The objectives of truthful disclosure of diagnosis to patients with dementia are to empower the patient with: the courage to request for information, the cognition to understand information and the strength and resources to cope with the burden of information<br><br>For many, the diagnosis of dementia can be devastating and thus, individuals with dementia and their family may have high, sometimes unrealistic, expectations of any treatments offered. It is therefore important to communicate from the onset that:<br>• The medications are not a cure.<br>• The medications may not be effective for everyone.<br>• Although there may be a response in terms of modest improvement or stabilization of symptoms, symptomatic therapy	long-term caregiving need to be helped and supported.<br><br>However, no one approach is necessarily sufficient to meet the varied needs and situations of individual caregivers. Therefore, multi-component interventions have a greater effect than narrowly focused ones. A support system that is tailored to the particular needs of different caregivers, and be able to provide on-going responsive and continuous support, is most beneficial.<br><br>Where appropriate,		Studies have shown that the vast majority of patients with mild dementia wish to be fully informed. Therefore, unless a patient suffering from dementia explicitly declines to be informed of the diagnosis, the default mode should be to inform truthfully as it will enable the patient to:<br>a. plan for optimal life experiences in remaining years of intact capacities<br>b. designate and appoint a surrogate decision maker (done of Lasting Power of Attorney as in the Mental Capacity Act) to take over the making of treatment decision upon eventual incompetence<br>c. settle personal financial and legal matters<br>d. participate in treatment decisions<br>e. consider possible enrolment in research programmes and participate in informed consent process<br>The decision-making process involving feeding options is often complex and involves taking in to	driving, doctors should initially enlist the help of family members to persuade patient to stop driving. To encourage such patients to surrender their driving licences, alternative forms of transport should be arranged, where possible.<br><br>Persons with mild dementia should be referred for a formal driving assessment.<br>(a) If deemed safe, they should be allowed to drive; in some instances, restrictions such as driving only when accompanied, driving only during daytime hours and not driving on expressways, may be recommended.<br>(b) They should be reassessed at least every 6-12 months depending on the recommendations of the driving assessment. Families and caregivers need to observe for any warning signs that may indicate unsafe driving. Whenever there is a change in status noted, considerations should be given for earlier formal driving assessment or in certain cases, cessation of driving.

		ultimately does not prevent progression of disease and cognitive decline will continue even with treatment.<br>• The medication may be discontinued if the patient does not demonstrate stabilization after an adequate trial of 3-6 months<br><br>In view of the potential adverse effects associated with antipsychotic therapy, non-pharmacological interventions and identification of pain and other environmental factors should be assessed and managed accordingly. If the above fails and the behavioural problems are assessed to be significant causing difficulty in caring process (medically and functionally) with significant amount of caregiver distress, there has to be a discussion with the family members with regards to antipsychotic therapy, with the attendant risks of adverse effects (extrapyramidal side effects, somnolence, stroke, metabolic complications), especially in those patients with risk factors for cerebrovascular disease.<br><br>The important question to ask for each individual patient is therefore what value of life holds for the patient, and directed at how life is experienced by the	respite care may be offered to relieve the burden of caregiving on caregivers of persons with dementia.<br><br>End-of-life care for patients with dementia has been found to be extremely demanding of family caregivers. The burden experienced can be personal, emotional and economic and can result in significant levels of depression in the caregivers. Therefore, care for patients with advanced dementia needs to address caregiver issues and provide adequate support to help them cope with difficulties experienced. Ac		account several issues such as advance directives, legal and financial concerns, religious and socio-cultural issues as well as emotive issues that revolve around the family caregiver's preferences. A careful consideration of these factors with involvement of the patient's family in discussions to reach shared decisions is necessary.<br>Advance care planning with regard to cardiopulmonary resuscitation (CPR) should be encouraged given the poor outcomes of CPR in advanced dementia	A diagnosis of dementia does not automatically mean that the patient is incapable of living alone. This decision should be based on an assessment of the patient's decision making capacity with respect to placement, and ability to continue living alone in the community without posing too much risk to self and to neighbours.<br><br>If the patient is assessed to have adequate decision making capacity and insists on living alone, the health care professionals should then support the decision by simplifying the daily tasks at home and using available community resources. The patient should also be reassessed as the dementia progresses and erodes both his decisional capacity and ability for self-care.

		patient under the contextual circumstances specific to the patient.<br><br>In general, the diagnosis of dementia should be disclosed to the patient, unless explicitly stated otherwise.<br><br>Although patients generally would like to know the truth about their own medical condition, the rights of those who do not want to know should also be respected. Health care professionals should therefore seek to understand their patients' preferences with respect to the diagnosis of dementia and act appropriately according to their choice.<br><br>When informing the diagnosis, the doctor needs to take into account the patient and family's prior knowledge and their perception of the problems.<br><br>Disclosure should not be a one-off event and must be seen as an ongoing, dynamic process and a fundamental part of the care of a patient with dementia.<br><br>They should be given ample opportunity to ask questions and seek clarification from the doctor.<br><br>The objectives of truthful	cess to the relevant support services should also be facilitated.			

		disclosure of diagnosis to patients with dementia are to empower the patient with: the courage to request for information, the cognition to understand information and the strength and resources to cope with the burden of information.<br><br>Caregivers of patients with young onset dementia should receive adequate counselling on the disease process, community resources and financial assistance.<br><br>Caregivers of persons with advanced dementia should be provided with adequate information on available community and medical resources				
Czech Republic 2010 (Hort et al., 2010)	--	The first step in AD management is accurate recognition and diagnosis of the disorder, and then disclosing that diagnosis in a sensitive and timely way to the patient and others as appropriate.<br><br>At time of diagnosis several issues need to be addressed, including the provision of high-quality understandable information about the illness and its course to patient and caregiver<br><br>Disclosure of diagnosis should be	Disclosure of diagnosis is not harmful, and actually decreases depression and anxiety in patients and their care-giver.<br><br>Care-giver stress and depression are common and, if present, more intensive care-giver support	In patients with AD, treatment with ChEIs (donepezil, galantamine, or rivastigmine) should be considered at the time of diagnosis, taking into account expected therapeutic benefits and potential safety issues. Benefits on cognitive and non-cognitive symptoms have been demonstrated in those with mild, moderate and severe disease. Realistic expectations for treatment effects and	Disclosure offers the patient opportunity to pursue desired activities and maximizes individual autonomy and choice by providing information necessary for decision making and advance planning, including the decision to give informed consent to research projects and autopsy.<br>If possible, physicians may encourage patients to draw up advance directives containing	--

		individually tailored. It should be accompanied by information and counseling, as well as useful contacts such as Alzheimer's patient organizations.	and counselling and/or specific treatment for depression may be needed.<br><br>Reassessment for development of co-morbidity (including carer stress) should be an integral part of management<br><br>Care-giver support should consist of education about AD, and attending peer support groups may be helpful.	potential side effects should be discussed with the patient and caregivers.<br><br>In patients with moderate to severe AD, treatment with memantine should be considered taking into account expected therapeutic benefits and potential safety issues. Benefits on cognitive and noncognitive symptoms are apparent, some non-cognitive symptoms (agitation, delusions) may respond better than others.<br><br>Realistic expectations for treatment effects and potential side effects should be discussed with the patient and caregivers.	future treatment and care preferences.<br>Low dose of atypical agents should be used only after assessment of risk benefit and full discussion with patient (when capacity allows) and caregiver.	
Spain 2010 (Ministry of Health Social Services and Equality, 2010)	--	Obtaining data from an independent and reliable informant is recommended, whenever possible.<br><br>Patients and/or their families must be informed about the diagnosis, the prognosis and the strategy to be followed.<br><br>Patients and/or their families must receive a written report listing all the examinations	It must be taken into account that the mental health services carry out an essential role in the care of people with dementia, as they contribute to the etiological	--	Coordination at the different healthcare levels is recommendable in order to favour respect for the rights and freedoms of patients in decision-making (advance directives).<br><br>Health and social services professionals, and relatives must try to discover the AD of sick	Favouring access to social services is recommendable as well as providing the services to give adequate support to the task of caring of families that care for people with dementia.<br><br>In each case and depending on the moment of evolution of the dementia, it is recommendable to dynamically select the best

		carried out, the diagnostic guideline and treatment, and monitoring plan. This information must be given to the PCT and/or to the referring practitioner.<br><br>It is recommendable for health and social services professionals to know about the problems that affect their dementia patients to be able to provide an answer to their demands and know what tasks caregivers must carry out, the time they spend, the environment where they live, and the cost it represents for the family.<br><br>Informing patients and their families of the dementia diagnosis is recommended when there is reasonable suspicion of dementia. The SDCT physician and/or PCT is the person who must inform the patient and family.<br><br>Informing patients who request this and the family of the diagnosis is recommended, as it has been proven that the information received does not predispose or generate a disorder in the person affected.<br><br>Offering information via understandable, sensible, respectful, empathetic, verbal communication, and an adequate environment and conditions is	diagnosis, the treatment and hospitalized care if severe BPSD appear. They provide coordinated, comprehensive and multidisciplinary care.<br><br>Psychological support and psychoeducational interventions geared towards families with people with dementia are recommendable, in order to favour their task of caring, reduce the care burden and improve the quality of life.<br><br>It is advisable for the group information sessions aimed at families to contemplate basic aspects of dementia; detection of burdens		people to be able to comply with their wishes and respect their medical, legal and financial preferences.<br><br>The utmost efforts should be made to maintain the autonomy of people affected by dementia, respecting their decision-making capacity, whenever possible, even at the end of their days.<br><br>It is advisable to involve the patient in decisions made about the treatment. The patient's desire must prevail; however, differences in opinion between patient and caregiver must be evaluated.	social service resource that must be provided to a family caring for a person with dementia.<br><br>The execution of specific training programmes for families who live with people affected by DLB, FTLD or VD and in advanced or terminal dementia stages is recommended.<br>It is recommendable to train caregivers (families or professionals) to empower them to manage non-pharmacological interventions of dementia.<br><br>Intervention programmes on activities of daily living are recommended to improve the functionality of patients with dementia and decrease the strain of the caregiver in the medium term, both in institutionalized people and those who live in their family homes.<br><br>It is especially important for caregivers to receive information and training that capacitates them to prevent the appearance of BPSD and act as co-therapists when they are already present.

		recommended, to facilitate two-way dialogue. To make it easier to understand the diagnosis and its consequences, further information can be provided via information leaflets, booklets, videos, Internet and other audiovisual means, and associations of relatives of dementia patients<br><br>Giving information to patients and families about dementia is recommended, as well as about its evolution, clinical manifestations, comprehensive treatment and support to the caregiver, which will enable them to plan their future<br><br>It is advisable for the group information sessions aimed at families to contemplate basic aspects of dementia; function of the different professionals and healthcare levels; comprehensive treatment and available resources; management of emerging problems	derived from the task of caring, and support to the caregiver.<br><br>The periodic assessment of cognitive, functional, motor and behavioural aspects is recommended, as well as of the degree of strain on the caregiver when monitoring patients with dementia.<br><br>During the care of every person with dementia, their individual characteristics and the actual characteristics of the disease must be evaluated to be able to assess the needs and prevent the burden of the caregiver, and act consequently to optimize their			It is advisable to inform and train caregivers of dementia patients in strategies to address and manage the BPSD so that it is possible to adopt and promote an appropriate attitude towards patients and prevent the strain and claudication of caregivers.<br><br>Caregivers should be informed well and progressively about the disease and its possible complications, offering them the available social resources, as well as the formal or informal support systems to prevent strain.<br><br>Psychotherapy, especially cognitive-behavioural therapy, would be recommended to treat stress or depression due to excess caregiver burden when they present clinically relevant symptomatology.

			support network<br><br>Dementia is a dynamic process, which, as it progresses, increases the commitment and obligations of the caregiver. It is recommendabl e to meticulously evaluate the burden supported by primary caregivers and the support they receive in their task of caring. If the burden is too much, it is advisable to establish the appropriate measures to prevent this from affecting their physical and psychological health, thus giving rise to the risk of claudication.			

			The use of the Zarit scale (long and short versions) is recommended to quantify the caregiver burden.<br><br>The use of specific adapted and validated instruments is recommended to assess other aspects that may be affected by the caregiver burden.<br><br>A basal evaluation of caregivers of people with dementia is recommendable in order to identify the factors that affect the burden of caring, the difficulties that might arise in the course of the disease and the degree of burden that			

			caregivers already have at the time of the diagnosis.<br><br>To improve the care of caregivers of people with dementia and reduce their burden in the task of caring, as well as anxiety and depression, a programme that combines educational support, emotional support and the provision of resources may be beneficial.<br><br>It is advisable and desirable for caregivers of people with dementia to participate in MAGs, as they provide many different benefits, which will enable them to cope better with the			

			task of caring and improve their personal situation.<br><br>Participation in TAGs or EESG to improve the strategies to cope with dementia is recommendabl e, relieving the burden and improving the level of anxiety and depression that might affect the caregiver.<br><br>It is recommended for those caregivers who express a significant degree of stress and/or depression to be referred to their primary care physician and/or mental health specialists for their assessment and treatment.			

			It is advisable to attend to the abuser, who is often a stressed or sick relative, or with an excessive burden. The burden can be reduced by sharing the task of caring, removing the patient from the family environment, participating in support groups, guaranteeing rest and offering medical treatment if required			
Malaysia 2009 (Ministry of health, Malaysia, 2009)	--	History from a reliable informant is mandatory<br><br>The family needs to be informed of the adverse events prior to commencement of treatment.<br><br>Assessment and care-planning approach<br>• Behavioural and functional analysis should be undertaken in conjunction with caregivers and care workers.<br>• Individual care plans should be developed and recorded in	ALGORITHM ON SCREENING OF DEMENTIA IN PRIMARY CARE<br>• Abnormalities detected on clinical assessment/ If No/ Provide reassurance<br>• Abnormalities detected on clinical assessment/ If	--	Promote Consumer-Directed Interventions. Caregivers who have more choice, control, and flexibility in their home care options are significantly more satisfied with overall service options.<br>Unless a patient suffering from dementia explicitly declines to know the diagnosis, the default mode should be to inform truthfully as it	Each patient should be assessed whereby a care plan is drawn to address issues, such as activities of daily living (ADLs) that can maximize independence, enhance function, adapt and develop skills, and to minimize the need for support.<br><br>Professionals trained in assessment and care planning can devise ADL skill training programmes

		patients' notes. These should be reviewed regularly with caregivers and other staff.<br><br>Information about the disease process, resources, services, and training on how to respond effectively to disease-related behaviours should be provided on a regular basis, preferably as a structured programme.<br><br>Although patients generally would like to know the truth about their own medical condition, the rights of those who do not want to know should also be respected. Health care professionals should therefore seek to understand their patients' preferences with respect to the diagnosis of dementia and act appropriately according to their choice<br><br>Patient's preference on the disclosure of the truth should be elicited.<br><br>Palliative care in dementia should begin from the time of diagnosis, up to the time of death. Communication about these issues should start as soon as possible, ideally at time when the diagnosis of dementia was made because individuals with dementia eventually lose the ability to make independent decisions about their future care.	Yes/ Perform laboratory test<br>• Any abnormalities present?/ If No/ Do the findings meet criteria for dementia?/If No/ Is the patient cognitively impaired but not demented/ If No/ Provide reassurance or referral as appropriate<br>• Any abnormalities present?/ /if Yes/ Treat and reassess □ Do symptoms remain?<br>- If Yes/ Do the findings meet criteria for dementia?/ If No/ Is the patient cognitively impaired but not demented/ If No/ Provide reassurance or referral as appropriate<br>- If No/ Provide reassurance		will enable the patient to:<br>a. Plan for optimal life experiences in remaining years of intact capacities<br>b. Designate and appoint a surrogate decision maker to take over the making of treatment decision upon eventual incompetence<br>c. Settle personal financial and legal matters<br>d. Participate in treatment decisions<br>e. Consider possible enrolment in research programmes and<br>f. Participate in informed consent process<br>Respect for autonomous decision making is a fundamental ethical and legal right of a mentally competent individual. This right of self-determination should be respected to the fullest possible extent, even in dementia.<br>People with dementia should be given the opportunity to convey information in a confidential manner while he or she still has capacity.<br>The PWD and their caregivers should be	for use by caregivers and/or care staff<br><br>Recommendation.<br>• A combination of interventions that promote communication, mobility and cognition is recommended to facilitate independence in these patients.<br>• Activities have to be individualized and adapted to maximize the person's remaining abilities.<br><br>Hence the management of dementia should also focus on helping the caregivers. A "one-size-fits-all" approach is deemed ineffective while specific interventions that are tailored towards the needs of the caregiver, implemented at the relevant trajectories of illness, would be most effective.<br><br>Training courses about dementia, services and benefits, communication and problem solving in the care of people with dementia

		Diagnosis disclosure-suggested guidelines<br>• Use a multi-professional approach to answer questions and make recommendations<br>• Allow each separate time to talk and ask questions<br>• Arrange follow-up meetings to continue discussions<br>• Discuss how the disease might progress<br>• Provide written educational materials<br>• Provide a list of community resources and contacts<br><br>Both PWD and their caregiver are entitled to receive relevant information regarding dementia, treatment, available support services, as well as legal, financial and benefits advice<br>This can be done through:<br>Written information<br>• Individual or group psychoeducation<br>• Telemedicine services<br>• Involvement of other family members as well as the primary caregiver in family meetings	Recommendati on.<br>• An evaluation of the caregivers' needs should be carried out on a routine basis.		educated on the use of advance statements (which allow people to state what is to be done if they should subsequently lose the capacity to decide or to communicate) and advance decisions to refuse treatment.<br>While the people with dementia still has the capacity, early consideration for advance statements and advance decision to refuse treatment should be planned.<br>When a people with dementia loses decision making capacity, it is important to protect the person from their own harmful decisions or actions.	
United States 2012 (Amante et al., 2012)	--	Often, using the term "Alzheimer's"—rather than "dementia"—is highly effective, because people have a general awareness of what that means and that there are next steps they need to take.	Sometimes patients and/or their family members are thinking about dementia but are afraid to ask about it. Many	Discuss age-related changes in memory, and validate that they are not necessarily a sign of disease.<br><br>Delivering a diagnosis of dementia: Talking points	Delivering a diagnosis of dementia- Talking points "It is important for you and your family to plan for the future, and it is especially important for you to make legal plans. The sooner legal	Goals of treatment for dementia- Midstage Dementia:<br>Develop skills that support continued living at home and delay institutionalization.

		Then discuss the role of acetylcholinesterase inhibitors. The drug part should be last, as it is not very effective and you do not want to create false hope. When these patients and their families are also dealing with behavior problems (i.e., argumentative, stubborn, yelling), behavioral skills for managing the problems and possibly using sedatives and antipsychotics may be discussed, too. But the key takeaways are: don't short-change diagnostics and conversations about care and safety, and do the drugs after.<br><br>The type of support the patient and family/caregiver need will evolve with the progression of the disease. The family should be aware that inevitable disease-related deficits will develop in memory, behavior, mood, and function (e.g., incontinence, immobility, confusion).<br><br>Family members should be made aware that behavioral symptoms can occur during phases of dementia and are not permanent<br><br>Conversation 3. Will medications help control behavioral symptoms of dementia? – Talking points<br>• "These medications have modest benefits in controlling behavior problems, but they have	times, though, the families are not aware that dementia is present, unless specifically asked questions about memory and function by the PCP.<br><br>The need for a PCP to lift the burden of worry about not doing enough is a key clinical task. The key take-away is to recognize that reassuring caregivers they are doing everything right—that they can let go of worry and guilt—is of high clinical value and is essential for good care for patients at this time.<br><br>Routine testing for genetic markers of medical conditions	• "Eventually, dementia will cause a worsening in your ability to handle regular tasks, such as shopping, finances, and medications. But we'll talk regularly, and we'll manage that."<br>• "Dementia is a progressive condition with no cure, but we have treatments for symptoms. And proper care and planning can greatly alleviate the burden of dementia."	planning starts, the more you may be able to participate. Legal planning includes advance directives."<br>Do I need to see a neurologist? - Key Considerations<br>In general, most patients do not need to see a neurologist, but Group Health neurologists are willing to see anyone who wants to be seen.<br>As part of the care plan, it is important for the patient and family/caregiver to establish advance directives as soon as possible<br>Medications for treating severe behavioral disturbances/safety risks<br>Evidence suggests that both escitalopram and citalopram are associated with QT interval prolongation starting at the recommended initial dose, with the strongest evidence existing for citalopram at doses exceeding 40 mg per day. Shared decision making is recommended to review the risks and benefits of these medications with the	 When considering treatment, also assess the impact of BPSD on the caregiver and the housing situation. If the patient's housing is in jeopardy because of BPSD, it is important to shift to a dual focus: patient centered and caregiver-centered interventions:<br>• Psychoeducation and mutual goals to maintain housing. or<br>• Consultation with Social Work to help with changing housing needs<br><br>Combination of memantine and acetylcholinesterase inhibitor (e.g., donepezil). During mid-stage dementia, memantine may be added to ongoing acetylcholinesterase inhibitor therapy with the intention of using the combined therapy to ease caregiver burden and to improve engagement of the patient with daily activities and social interactions.<br><br>Options for treating mild to moderate behavioral disturbances<br>Wandering (leaving the house)<br>• Keyed deadbolt for entry

		severe side effects."<br>• "If the behavioral symptoms are impacting your loved one's current housing situation, these medications may not help. A social worker may be able to help you with your housing issues."<br>• "Medications are a short-term intervention that must be regularly re-evaluated."<br><br>Medication monitoring for patients with dementia- Assessment<br>• Ask patient and caregiver/family about medication effectiveness and side effects.<br>• Assess whether treatment goals have been met.	known to cause dementia, such as Huntington's chorea, is not recommended because false positives may occur and would be emotionally and financially devastating.<br><br>In determining a patient's behavioral issues, PCPs may find it helpful to use a caregiver survey such as the Revised Memory and Behavioral Problems Checklist. It addresses the frequency of different types of behaviors and how troubling the behaviors are to the caregiver.<br><br>Progression monitoring for patients with dementia and		patient.<br>If you are using medications, it is not unreasonable to start at diagnosis, provided you do the following:<br>Develop functional and/or behavioral goals with the patient and family/caregiver to help assess whether the medications are providing benefit.<br>Patient goals might include managing a checkbook, maintaining a prescribed medication schedule, increasing social interaction; family/caregiver goals might include improving quality of life and facilitating the ease of caregiving<br>Genetic testing might be helpful when results would affect treatment decisions for the patient or reproductive decisions by younger family members	to and exit from the house.<br>• Lock box for keys. ID bracelet and/or picture ID.<br>• MedicAlert® + Alzheimer's Association Safe Return® program, a nationwide emergency response service<br>Refusing care/medication<br>• Explore "keys" to unlock cooperation (e.g., activities the patient enjoys, or finds calming or distracting).<br>• Discuss with family hiding medication in food.<br><br>Table 13. Progression monitoring for patients with dementia and for caregivers/families<br>Assess caregiver's ability to manage care:<br>• Is there continued understanding of what caregiver needs to know and/or have to care for patient?<br>• Are there any new or different services or resources available to help caregiver?<br>Address behavioral problems and safety concerns:<br>• Verbal/physical outbursts.<br>• Wandering.<br>• Refusing care/medication.

			for caregivers/families<br>Evaluate mood:<br>• How is the patient feeling?<br>• How is the caregiver feeling?<br><br>A referral to Genetics is not likely to be helpful for first-degree relatives of individuals whose dementia onset occurred after age 60, as there are no specific genetics tests to be done. Concerned family members might find the Family and Genetics page on the Alzheimer's Association website to be reassuring.<br><br>Being a caregiver for a patient with dementia is			• Driving.<br><br>Referral to a specialist for diagnostic consultation and a treatment plan to be followed in primary care may be considered if any of the following criteria apply:<br>Social Work<br>Documented diagnosis of dementia with concerns about safety, behavioral problems, or housing.<br><br>Tips for caregivers on dealing with behaviors<br>Dealing with verbal and physical outbursts<br>• Remain calm. Be reassuring and positive. Speak slowly and in a soft tone.<br>• Consider what might be contributing to the patient's behavior. Is he/she tired, overstimulated by noise or an overactive environment, or picking up on your own stress or irritability?<br>• Rule out pain as the cause.<br>• Think about what happened right before the behavior that may have triggered it.<br>• Try a relaxing activity, or shift to a different activity—the immediate situation may have unintentionally

			extremely difficult. Remind caregivers that their own health is important. Give caregivers reassurance that they are doing a good job and that they are doing everything they can do.<br><br>Assess caregivers for continued ability to care for the patient and to ensure that their own health is not being compromised.<br><br>Refer caregivers to Social Work or Behavioral Health, or to a community resource:<br>• If they are experiencing fatigue, depression, anxiety, anger, or other stress-related signs.			caused the response.<br>• Decrease level of danger. Avoid harm to yourself by standing away from the patient.<br>• See the Aggression and Anger page on the Alzheimer's Association website<br>Dealing with wake/sleep disturbances<br>• Make a safe and comfortable sleep environment (i.e., temperature, nightlights, appropriate door/window locks).<br>• Maintain a schedule. A regular routine of waking up, meals, and going to bed allows for more restful sleep.<br>• Identify and limit triggers—such as TV, loud music—especially during evening hours.<br>• See the Sleep Issues and Sundowning page on the Alzheimer's Association website<br>Dealing with unsafe driving<br>• Acknowledge patient's distress and threat to independence.<br>• Ensure safety of patient and others.<br>• Consider a professional driving assessment, if there is family conflict over the issue.

						• Create an action plan to prevent unsafe driving, because this will worsen over time.<br>• See the Dementia & Driving Resource Center on the Alzheimer's Association website
Australia 2008 (Abbey et al., 2008)	--	Health professionals are responsible for:<br>• Providing support and information for the person with dementia and their carer regarding the diagnosis of dementia<br>• Understanding the knowledge of the person with dementia and their carer with respect to the diagnosis of dementia and its implications<br><br>Carers, as informed sources, are a good source of reported decline in a person's cognitive function. If loss of cognitive function is suspected by a carer, the health professional should assist the carer to complete the Informant Questionnaire on Cognitive Decline in the Elderly (IQCODE) (18) or the Kimberley Indigenous Cognitive Assessment (KICA) - Carer (19), a sub component of the KICACog (19), which may be more culturally appropriate for specific indigenous Australians.<br><br>Assessment and diagnosis: this assessment should include a medical, family, social, cultural	Family members may also benefit from referral to a clinical psychologist for psychotherapy and social workers can assist with counselling and linking to community resources.<br><br>Differential diagnosis<br>The Neuropsychiatric Inventory Questionnaire, short format (NPI-Q) (39) may be useful in assessing neuropsychiatric symptoms including apathy depression, psychosis and agitation as	--	The principles that underline the National Framework for Action on Dementia and inform the development of these Guidelines and Pathways are:<br>People with dementia, their carers and families are central to making choices about care<br>These Clinical Practice Guidelines (Guidelines) and Care Pathways (Pathways), as part of the Cognition/Dementia Care System, promote the adoption of these principles in practice and also incorporate the following:<br>Consultation and collaboration with people who have dementia, their carers, Health professionals and advocates is essential to developing and delivering good dementia care<br>In early dementia the person usually has the	Maintenance of function: Health care practitioners can assist the person with dementia and their carer to promote and maintain independence. Care plans should seek to maximize independent activity, enhance function, adapt and develop skills and minimize the need for support.<br><br>Care managers should ensure that people with dementia and their carers have access to a comprehensive range of respite/short break services. This could include centre based day care, in home day and over-night respite, short term cottage respite, or overnight care in residential settings. Respite in a Residential Aged Care Facility (RACF) normally requires approval from an Aged Care Assessment Team (ACAT). Health professionals should assist the carer in forward

		and medication history and detail of the chief complaint<br><br>Following a diagnosis of dementia, clinicians should give adequate time and an appropriate opportunity to discuss the diagnosis and its implications with both the person with dementia and family members<br><br>- Health professionals need to inform the family of the diagnosis of dementia<br>- Practitioners need to provide the person and their family with written information about: signs and symptoms of dementia, the anticipated course and prognosis, treatments, local care and support services, support groups, financial, legal and advocacy advice, and voluntary organizations. Any advice given needs to be clearly documented in the person's notes<br><br>Post Diagnosis, Monitoring, Management And Care Phase<br>Specifically in this phase health professionals are responsible for:<br>• Establishing information sharing and communication between care providers<br>• Providing effective communication strategies for the person with dementia, their carer and care providers<br>• Assessing the carer's	well as related carer concerns. The scale can assist in identifying different causes of dementia and including symptoms, which are often associated with fronto-temporal dementias. It can also be helpful in assessing the level of caregiver distress.<br><br>Guideline<br>The Neuropsychiatric Inventory Questionnaire (NPI-Q) can be useful to assess neuropsychiatric symptoms and the impact this has on the caregiver. It assists in identifying different causes of the dementia<br><br>The Neuropsychiatri		capacity to identify a surrogate decision maker. Legal matters and decisions need to be addressed soon after diagnosis while the person who has dementia is still able to make and communicate these decisions<br>In some families of a CALD background a key decision maker makes all the decisions surrounding the care of the person who has dementia and how other family members should respond. It is important to identify this person so as to inform their decisions and to respond to the needs of all involved family members. The primary carer may not have decision making authority within their family<br>Post Diagnosis, Monitoring, Management And Care Phase<br>Specifically in this phase health professionals are responsible for:<br>• Establishing goals aimed at maintaining independence, self-care and safety, with the	planning and be aware that assessment and approval by an ACAT should be planned in advance of the need for residential respite (22) wherever possible. Flexible transport may be required if full access to these services is to occur. Respite or short break care needs to be meaningful, tailored and in an environment that meets the needs of the person with dementia and their carer.

		knowledge and role in providing support for the person with dementia<br>• Providing information and support to both the person with dementia and their carer<br><br>Maintenance of function<br>It is suggested that regular health assessments incorporate assessment of the skills required for safe driving as well as a discussion of any indicators that driving is becoming unsafe<br><br>Driving ability may be impaired and the person with dementia should be advised of how their condition may impact on their driving and of the need to report their diagnosis to the driving licensing authority and to their insurer<br><br>Service provision<br>The carer and the person who has dementia need to be provided with advice on how agencies work together to provide a comprehensive service from diagnosis to the advanced phase<br><br>The carer and the person with dementia need to be provided with advice on how agencies work together to provide a comprehensive service from diagnosis to the palliative phase	c Inventory Questionnaire NPIQ short format can be used to assess neuropsychiatric symptoms and caregiver distress<br><br>A palliative approach:<br>Feelings of grief and loss need to be anticipated from the time of diagnosis to death and grief and bereavement counselling should be available to carers.<br><br>Symptom management<br>Health professionals caring for clients with dementia need to be knowledgeable about pain assessment and management in this population		person who has dementia and their carer<br>• Establishing procedures for regular review of care goals and developing new goals as required<br>• Reviewing advance care directives<br>• Supporting the carer in making decisions regarding alternative care arrangements when appropriate<br><br>Advanced Phase<br>In this phase health professionals are responsible for:<br>Liaising with carers regarding advance health directives particularly with regard to nutrition, hydration, symptom management and place of care<br>Admission to Residential Care<br>Health professionals need to assist the carer in forward planning and be aware that assessment and approval by an ACAT needs to be planned in advance of the need for residential care.<br>Where care in a setting other than the home is	

		Impact of caring on sexual relationships<br>Health professionals' awareness of the impact of dementia on the intimate and sexual relationship between the carer and the care recipient, and an ability to be open to discussion about this, is helpful.	so that physical and emotional well-being is promoted		required, staff awareness of the wishes of the person who has dementia with regard to life sustaining treatments allows for their personal choices to be honoured.<br>7.2.1 Decision making/advance directives<br>While decisions regarding advance directives may have been discussed at diagnosis and throughout the progression of dementia, during the advanced phase it is important for health professionals to review these decisions with carers.	
United States 2008 (California Workgroup on Guidelines for Alzheimer's Disease Management, 2008)	Establishing and maintaining alliances with caregivers is critical for care of the Alzheimer's Disease patient.<br><br>Major physician organizations have	It is important to recognize that the needs of people with Alzheimer's Disease and their families extend far beyond the realm of medical treatment, and that PCPs will be called upon to provide a wide spectrum of information and resources to assist them in dealing with this challenging, sometimes overwhelming condition.<br><br>Develop and implement an ongoing treatment plan with defined goals. Discuss with patient and family:	However, it is important to recognize that the needs of people with Alzheimer's Disease and their families extend far beyond the realm of medical treatment, and that PCPs will be called upon to provide a	There is a tension between the patient's right to autonomy and the caregiver's duty to protect. The PCP should assess and assist with the need for balancing these concerns with respect to such decisions as determining the time to stop driving.	Assess Capacity. Assess the patient's decision-making capacity and determine whether a surrogate has been identified.<br>Involve Early-Stage Patients. Pay particular attention to the special needs of early-stage patients, involving them in care planning, heeding their opinions and wishes, and referring them to community resources, including the	Identify Support. identify the primary caregiver and assess the adequacy of family and other support systems, paying particular attention to the caregiver's own mental and physical health.<br><br>Early-Stage Recommendations. Discuss implications with respect to work, driving, and other safety issues with the patient. recommend interventions to protect and

	emphasized the importance of family caregivers by calling on PCPs to form partnerships with families who care for dementia patients.<br><br>Management goals and interventions should be based on a solid alliance with the patient and family and on thorough psychiatric, neurological, and general medical evaluations of the nature and cause of cognitive deficits and associated noncognitive symptoms.	Use of cholinesterase inhibitors, NMDA antagonist, and other medications, if clinically indicated, to treat cognitive decline.<br><br>Discuss Diagnosis & Treatment. Discuss the diagnosis, progression, treatment choices, and goals of Alzheimer's Disease care with the patient and family in a manner consistent with their values, preferences, culture, educational level, and the patient's abilities.<br><br>The assessment should also address the patient's support system and decision-making capacity, and identify the primary caregiver who, in addition to other family members, is a critically important source of information.<br><br>Assessment: Comorbid Medical Conditions. The involvement of family members and other caregivers in gathering a history and completing an evaluation to identify co-morbid medical conditions is essential<br><br>The family is an excellent source of information regarding a patient's baseline level of functioning. This will assist the PCP in determining whether there is an acute medical condition in addition to	wide spectrum of information and resources to assist them in dealing with this challenging, sometimes overwhelming condition.<br><br>Once these potential issues are addressed, assessment should focus on the frequency, severity, and duration of particular behaviors as well as caregiver stress and coping strategies.<br><br>The Neuropsychiatric Inventory Questionnaire (NPIQ) is a brief, reliable, informant-based assessment of neuropsychiatric symptoms and associated caregiver distress and is		Alzheimer's association. Discuss Stages. Discuss the patient's need to make care choices at all stages of the disease through the use of advance directives and identification of surrogates for medical and legal decision-making.<br>Discuss End-of-Life Decisions. Discuss the intensity of care and other end-of-life care decisions with the Alzheimer's Disease patient and involved family members while respecting their cultural preferences.<br>Planning. include a discussion of the importance of basic legal and financial planning as part of the treatment plan as soon as possible after the diagnosis of Alzheimer's Disease.<br>Early-Stage Recommendations. Pay particular attention to the special needs of early-stage patients, involving them in care planning and referring them to community resources.<br>The Primary Care Practitioner (PCP) should	promote continuing functioning, assist with independence, and maintain cognitive health including physical exercise, cognitive stimulation and psychosocial support.<br><br>Recommend: interventions to protect and promote continuing functioning, assist with independence, and maintain cognitive health including physical exercise, cognitive stimulation and psychosocial support.<br><br>Careful and competent functional assessment enables the PCP and family to determine how best to maximize patients' independence.<br><br>Recommendations<br>• Conduct and document an assessment and monitor changes in daily functioning, including feeding, bathing, dressing, mobility, toileting, continence, and ability to manage finances and medications.<br>• Identify the patient's and family's culture, values, primary language, literacy level, and decision-making process.

		Alzheimer's Disease.<br><br>The PCP should request information from the caregiver about any other medical care received.<br><br>Standardized tools can be used by PCPs or clinic staff to gather information on behavioral symptoms from the caregiver and evaluate effectiveness of interventions over time.<br><br>As Alzheimer's Disease progresses, collateral information from the caregiver becomes essential to diagnose, treat and track the course of patients' depressive symptoms, and to monitor patients' suicidal potential. The Cornell Scale for Depression in Dementia is a useful tool for providers because it captures both patient and caregiver input.<br><br>The PCP must rely on family members to report relevant information. Therefore, the PCP should routinely solicit and incorporate family and other caregivers' reports of patients' changes in daily routine, mood, behavior, sleep patterns, weight gain or loss, and gait and mobility.<br><br>Assessment: Language, Culture, and Literacy	appropriate for use in a general clinical practice.<br><br>Thus, caregiver assessment should seek to identify any psychological distress as well as the psychological impact upon the caregiver with respect to changes in the cognitive status or behavior of the Alzheimer's Disease patient receiving care.<br><br>Caregivers should continue to be assessed even if the decision for long-term placement (e.g., nursing home) has been made because there is strong evidence that many caregivers continue to provide care		solicit and consider caregiver and family input in post-diagnostic treatment planning.<br>Assess the patient's decision-making capacity and determine whether a surrogate has been identified.<br>Caregiver assessment should:<br>• result in a plan of care, developed collaboratively with the caregiver, that identifies services to be provided and intended measurable outcomes<br>Assessment: Capacity Determination and Surrogate Identification<br>The PCP should determine decision-making capacity at the initial assessment and should ask the patient and family whether a surrogate decision-maker has been identified by the patient. The patient who has the capacity to identify a surrogate should be encouraged to do so as soon as possible for the sake of improving the quality of care over the course of the illness<br><br>Recommendation:	Caregivers may be able to assist at home by keeping a log of troubling behaviors that includes the times they occur, as well as strategies that are successful in modifying or curtailing these symptoms.<br><br>Regular appointments allow the PCP to monitor the patient's cognitive and functional status, as well as the development and evolution of cognitive and behavioral symptoms of Alzheimer's Disease and their response to intervention. They also provide a forum for health promotion and maintenance activities (Dunkin & Anderson-Hanley, 1998) and an opportunity to assess how well the caregiver is managing.<br>Recommendations<br>Develop and implement an ongoing treatment plan with defined goals. Discuss with patient and family:<br>Referral to social service agencies or support organizations, including the Alzheimer's Association's MedicAlert® + Safe Return® program for patients who

		Third, PCPs need to tap into underlying belief systems regarding Alzheimer's Disease and other comorbid conditions. This underlying world view and accompanying normative expectations are often expressed in terms of "folk understandings" which may influence the way in which people from diverse cultures receive and act on the information and directions provided by the PCP.<br><br>The PCP should make sure that the caregiver's contact information is noted and kept up to date in the patient demographics section of the patient's medical record<br><br>With respect to Alzheimer's Disease management, assessment of health literacy should focus on both the patient (in the early stages) and the primary caregiver (in all disease stages). Caregiver health literacy is especially critical as patient care responsibilities shift from the patient to the caregiver with disease progression.<br><br>Treatment: Developing a Treatment Plan (Therapies for Cognition)<br>PCPs should counsel patients with Alzheimer's Disease and their families about realistic	after placement, and the effects of caregiver strain and burden may still be present<br><br>Recommendation: Identify the primary caregiver and assess the adequacy of family and other support systems, paying particular attention to the caregiver's own mental and physical health.<br><br>The PCP must address caregiver support on an ongoing basis, and assess caregivers' mental and physical health regularly.<br><br>Effective interventions include assessing caregivers for depression and		Assess the patient's decision-making capacity and determine whether a surrogate has been identified.<br>Assessment: Language, Culture, and Literacy<br>Second, PCPs must be able to understand the patient's and family's customary ways of relating to others within their own group and with persons in authority, being aware that internal decision-making processes may vary both among and within different cultural groups. For example, the PCP may be seen as the sole person in authority, with the expectation that he or she will be making detailed caregiving decisions.<br>PCPs must ascertain as early as possible in the assessment process how a family makes decisions and identify its primary decision-maker, who may not be the person doing most of the "hands-on" caregiving.<br>Effective treatment requires development and implementation of a plan with defined goals for the patient. Goals	may wander.<br><br>To successfully navigate the challenging and unpredictable course of Alzheimer's Disease, patients and their families need a variety of community-based and long-term care resources as a complement to PCP care. Such services range from legal and financial planning early in the disease to skilled nursing care and hospice at the end of life<br><br>Recommend the following non-pharmacological interventions (preferably in combination) to protect and promote continuing functioning, assist with independence, and maintain cognitive health:<br>6. Driving evaluations at least every 6 months, including an on-road test with an experienced driving specialist<br>7. individualized instruction in activities to promote independence (e.g., cell phone usage, computer e-mail programs, etc.)<br>8. electronic reminder and monitoring programs (if not cost-prohibitive).

		expectations of treatment outcomes with these agents, which are likely to be small.<br><br>The agents are approved for monotherapy as well as combination therapy to improve cognitive function or delay decline in patients with mild, moderate, or severe dementia. PCPs should counsel patients with Alzheimer's Disease and their families about realistic expectations of treatment outcomes with these agents, which are likely to be small<br><br>Recommendations: Develop and implement an ongoing treatment plan with defined goals. Discuss with patient and family the use of cholinesterase inhibitors, NMDA antagonist, and other medications, if clinically indicated, to treat cognitive decline.<br><br>PCPs should take the extra time to explain possible benefits and side effects and establish criteria on which to base a decision for continuation.<br><br>Common Alzheimer's Disease-Related Behavioral Symptoms and Their Treatment. Wandering Caregivers should be advised that wanderers burn extra calories, so additional snacks may need to be provided to decrease the risk of	perceived burden and offering psychopharmacological treatment, supportive psychotherapy, support and education groups, and respite services to alleviate caregiver burden.		should be developed in consultation with the patient (if capable) and with the patient's family, using an individualized approach to their needs, values, and preferences, and should be modified as the disease progresses.<br>Early discussion of future care options with the patient and family will provide guidance to the Primary Care Practitioner (PCP) in modifying patient care goals over time in ways that is acceptable to patients with Alzheimer's Disease and their family members.<br>Patients and their families should participate fully in the decision-making process, and individual decisions should be based on clear understanding of the probable benefits and risks of therapy and personal patient preferences.<br>Pay particular attention to the special needs of early-stage patients, involving them in care planning, heeding their opinions and wishes, and referring them to	

		weight loss.<br><br>Depression<br>Collateral information from the caregiver is essential in diagnosing behavioral symptoms such as depression, and the PCP may find the Cornell Depression Scale for Depression in Dementia (Alexopoulos, Abrams, Young, & Shamoian, 1988) (see Appendix E), which includes caregiver input, to be a useful tool in diagnosing and treating major depression and monitoring suicidal potential.<br><br>Pharmacologic treatment of sleep disorders must take into account whether depressive symptoms, fear, pain, or side effects from other drugs underlie the insomnia (Warshaw et al., 1995). Great caution must be exercised and caregivers warned because of the possibility of reactions to major tranquilizers, which may include incontinence, instability and falls, and agitation.<br><br>Discuss the diagnosis, progression, treatment choices, and goals of Alzheimer's Disease care with the patient and family in a manner consistent with their values, preferences, culture, educational level, and the patient's abilities.<br><br>Caregiver Education			community resources, including the Alzheimer's Association.<br>Discuss the patient's need to make care choices at all stages of the disease through the use of advance directives and identification of surrogates for medical and legal decision-making<br>Discuss the intensity of care and other end-of life care decisions with the Alzheimer's Disease patient and involved family members while respecting their cultural preferences.<br>Moderate evidence suggests that there is a lack of knowledge and understanding of end-of-life care among the general population. Therefore, it is important for the PCP to discuss end of-life treatment goals and options with patients and families early on (Kettl, 2007). End-of-life treatment options and decisions need to take into account effective pain management, the goals of the patient (via advance directive), and patient and caregiver	

		The PCP should provide information and education about the current stage of the disease process and talk with the patient and family to establish treatment goals.<br><br>Based on the agreed-upon goals, a discussion regarding the expected effects (positive and negative) of interventions on cognition, mood, and behavior will ensure that the prescribed treatment strategy is appropriate to family values and culture.<br><br>General Legal and Financial Advice<br>The PCP also plays a critical role in providing guidance to the family regarding the need for financial and legal advice (Ham, 1997; Lyketsos et al., 2006). Efforts should be made to get the patient and family to seek sound professional advice (Overman & Stoudemire, 1988). Recommendations should include consultation with financial advisors and legal counsel and discussion of conservatorship.<br><br>Recommendation: Integrate medical care with education and support by connecting patient and caregiver to support organizations for linguistically and culturally appropriate educational materials and referrals to community			satisfaction.<br>The PCP should provide sufficient information so that reasonably informed decisions can be made with respect to medical treatment and other matters.<br><br>Advance directives and designation of healthcare surrogates should be put in place early, while the patient can still have input.<br>The PCP should also discuss values, preferences, and goals related to death and dying with patients in early stages of Alzheimer's Disease, including do not-resuscitate orders, artificial nutrition plans, and healthcare proxies.<br>Expert opinion and Workgroup consensus suggest that PCPs should initiate conversations with patients and their families about late-stage care and appointing a proxy.<br>PCPs need to respect the decisions of patients and their proxies, even though their cultural beliefs or wishes may be counter to medical	

		resources, support groups, legal counseling, respite care, consultation on care needs and options, and financial resources.<br>• Organizations include: Alzheimer’s Association<br>• Caregiver Resource Centers<br>• or your own social service department.<br><br>Patient and Family: Disclosure of Diagnosis and Family Conferences.<br>It is important that disclosure of the diagnosis of Alzheimer’s Disease be handled in accordance with the wishes of the patient and family.<br><br>Patient and Family: Disclosure of Diagnosis and Family Conferences<br>The consensus opinion of experts involved with the diagnosis and management of Alzheimer’s Disease is that a meeting with the patient and supportive family member(s) should be held when disclosing the diagnosis, allowing enough time for the PCP to discuss recommendations and to answer questions.<br><br>Ideally, a follow-up session should be scheduled to continue discussion since the information may be overwhelming at first, and patients and their families will have more questions over time.			recommendations.<br>Discuss the patient’s need to make care choices at all stages of the disease through the use of advance directives and identification of surrogates for medical and legal decision-making<br>Include a discussion of the importance of basic legal and financial planning as part of the treatment plan as soon as possible after the diagnosis of Alzheimer’s Disease.	

		If a key family member is unable to attend a face-to-face information session regarding disclosure of the diagnosis, the disease prognosis, treatment alternatives, and expected treatment outcomes, the PCP needs to identify and communicate with that person.<br><br>Also, for those patients who do not have family, the PCP should identify other members of the patient's informal support system who may be able to provide relevant history and observations or be enlisted to help monitor the patient's treatment plan recommendations, pending his or her consent and release for communication.<br><br>Patient and Family: Disclosure of Diagnosis and Family Conferences<br>It is often difficult for family members to discuss critical health care decisions. Strategies for PCPs to assist families in discussing these decisions include:<br>(a) initiating a discussion of goals for treatment to encourage families to talk about difficult choices in advance;<br>(b) enhancing the patient's and family's knowledge and understanding of health care procedures and care options so				

		that caregivers can ask more informed questions and better assess information they receive from health care professionals at different stages of the Alzheimer's Disease process; (c) helping families develop successful problem-solving strategies; and<br><br>Recommendation: Discuss the diagnosis, progression, treatment choices, and goals of Alzheimer's Disease care with the patient and family in a manner consistent with their values, preferences, culture, educational level, and the patient's abilities.<br><br>Recommendation: Include a discussion of the importance of basic legal and financial planning as part of the treatment plan as soon as possible after the diagnosis of Alzheimer's Disease.				
United States 2007 (Rabins et al., 2007)	The treatment of patients with dementia should be based on a thorough psychiatric, neurological, and general medical evaluation	At each stage the psychiatrist should be vigilant for symptoms likely to be present, should identify and treat co-occurring psychiatric and medical conditions, and should help patients and families anticipate future symptoms and the care likely to be required<br><br>In order to offer prompt treatment, enhance safety, and provide timely advice to the patient and family, it is generally	Because family members are often responsible for implementing and monitoring treatment plans, their own attitudes and behaviors can have a profound effect on the patient, and they often	Educate the Patient and Family About the Illness and Available Treatments<br>Family members and other caregivers may be particularly concerned about behavioral and neuropsychiatric symptoms, which they often associate with a loss of dignity, social stigma, and an increased caregiving	Patients and family members should be offered the opportunity to discuss preferences about participation in research studies early in the course of the illness, while the patient is still able to make his or her wishes known<br>When a patient's capacity is diminished but still sufficient to give consent, consent or at	Finally, other key tasks include providing critical support for family members and other caregivers and making referrals to social, legal, and other community resources.<br>All patients and families should be informed that even mild dementia increases the risk of vehicular accidents [I].<br>Mildly impaired patients should be advised to limit

	of the nature and cause of the cognitive deficits and associated noncognitiv e symptoms, in the context of a solid alliance with the patient and family.<br><br>Establish and Maintain an Alliance with the Patient and the Family. As with any psychiatric care, a solid therapeutic alliance is critical to the treatment of a patient with dementia. The care of a patient with dementia requires an alliance with the patient,	necessary to see patients in routine follow-up at least every 3–6 months<br><br>Important aspects of psychiatric management include educating patients and families about the illness, its treatment, and sources of additional care and support (e.g., support groups, respite care, nursing homes, and other long-term-care facilities) and advising patients and their families of the need for financial and legal planning due to the patient's eventual incapacity (e.g., power of attorney for medical and financial decisions, an up-to-date will, and the cost of long-term care) [<br><br>Patients and families should be advised about potential benefits and risks of antipsychotic agents, particularly the risk of mortality<br><br>Therefore, in order to offer prompt treatment, enhance safety, and provide timely advice to the patient and family, it is generally necessary to see patients, usually together with their caregivers, at regular follow-up visits.<br><br>Establish and Maintain an Alliance with the Patient and the Family.<br>Family members and other caregivers are a critical source of	need the treating physician's compassion and concern. For these reasons, treatment is directed to the patient-caregiver system.<br><br>Family members often feel overwhelmed by the combination of hard work and personal loss associated with caring for an individual with dementia. The caring and pragmatic attitude of the psychiatrist may provide critical support. This attitude may be expressed through thoughtful inquiries about current needs and how they are being met,	burden. It may be helpful to reassure patients and their families that these symptoms are part of the illness and are direct consequences of the damage to the brain.<br><br>Moreover, they may be relieved to know that although cognitive losses are generally not reversible, neuropsychiatric symptoms, especially the more disruptive ones, can often be improved or even eliminated with treatment, resulting in an overall increase in functional status and comfort.<br><br>Family members may differ in their opinion of the patient's level of functioning and may have different psychological responses to the patient's impairments, generating family conflict. It may be beneficial to meet with family members to openly discuss these issues.	least agreement is usually obtained from both patient and family member<br>Patients should be advised to complete or update their wills while they are able to make and express decisions. Patients and families should also be advised of the importance of financial planning early in the illness. This advice may include a frank discussion regarding the financing of home health care and/or institutional care	their driving to safer situations or to stop driving [I], and moderately impaired patients should be instructed not to drive [I]. Advice about driving cessation should also be communicated to family members, as the implementation of the recommendation often falls on them [I].<br><br>Consequently, when using pharmacotherapy in patients with dementia, low starting doses, small increases in dose, and long intervals between dose increments may be needed, in addition to ensuring that a system is in place that can enhance proper medication adherence [I]<br><br>The decision to remain at home should be reassessed regularly, with consideration of the patient's clinical status and the continued ability of the patient's caregivers to care for the patient, manage the burden of care, and utilize available support services.<br><br>Provide Education and Support to Patients and Families<br>a. Educate the Patient and

	as well as with the family and other caregivers.	information, as the patient is frequently unable to give a reliable history, particularly as the disease progresses.<br><br>Perform a Diagnostic Evaluation and Refer the Patient for Any Needed General Medical Care.<br>In general, many elements of the history will need to be obtained from the caregiver or the documented medical record as well as from the patient<br><br>Before undertaking an intervention, the psychiatrist should enlist the help of caregivers in carefully characterizing the target symptoms. Their nature, intensity, frequency, precipitants, and consequences should be reviewed and documented. This process is critical to revealing the cause of the symptoms, as well as monitoring the impact of any intervention. This approach also assists caregivers in beginning to achieve some mastery over the problematic symptom. Before embarking on any intervention, it is also helpful if clinicians explicitly review their own, the patient's, and the caregivers' expectations.<br><br>Caregivers should be referred to available books that provide advice and guidance about maximizing the safety of the	advice about available sources of emotional and practical support, referrals to appropriate community resources, and supportive psychotherapy.<br><br>Psychiatrists caring for patients with dementia should be vigilant for these conditions in caregivers, because they increase the risk of substandard care, neglect, or abuse of patients and are a sign that the caregivers themselves are in need of care<br><br>When a caregiver is in significant distress, his or her need for greater			Family About the Illness and Available Treatment<br>The family should be educated regarding basic principles of care, including<br>1) recognizing declines in capacity and adjusting expectations appropriately,<br>2) bringing sudden declines in function and the emergence of new symptoms to professional attention,<br>3) keeping requests and demands relatively simple,<br>4) deferring requests if the patient becomes overly upset or angered,<br>5) avoiding overly complex tasks that may lead to frustration,<br>6) not confronting patients about their deficits,<br>7) remaining calm, firm, and supportive and providing redirection if the patient becomes upset,<br>8) being consistent and avoiding unnecessary change, and<br>9) providing frequent reminders, explanations, and orientation cues.<br><br>Moderately Impaired Patients<br>Families should be counseled to undertake measures to prevent patients from driving, as

		environment for patients with dementia<br><br>Risks of driving should be discussed with all patients with dementia and their families, and these discussions should be carefully documented. Discussions should include an exploration of the patient's current driving patterns, transportation needs, and potential alternatives. The psychiatrist should also ask the family about any history of getting lost, traffic accidents, or near accidents. For patients with dementia who continue to drive, the issue should be raised repeatedly and reassessed over time.<br><br>Psychiatrists should familiarize themselves with state motor vehicle regulations for reporting individuals with dementia. In some states, disclosure is forbidden. In others, a diagnosis of dementia or Alzheimer's disease must be reported to the state department of motor vehicles, and the patient and family should be so informed.<br><br>Educate the Patient and Family About the Illness and Available Treatments<br>Terms should be clarified at the outset to facilitate	psychosocial support should be evaluated. If treatment is indicated, it can be provided (according to the preference of psychiatrist, patient, and caregiver) by the patient's psychiatrist or through a referral to another mental health professional.<br><br>Moderately Impaired Patients<br>As a patient's dependency increases, caregivers may begin to feel more burdened. A referral for some form of respite care (e.g., home health aid, day care, brief assisted living, or nursing home stay) may be helpful.			many patients lack insight into the risk that their continued driving poses to themselves or others.

		communication. Patients vary in their ability and desire to understand and discuss their diagnosis. Most mildly and some moderately impaired individuals are able to discuss the matter at some level, but the discussion must be adapted to the specific concerns and abilities of the patient; it may be helpful to seek the family's input regarding the nature and timing of any discussion with the patient.<br><br>Decisions about how to disclose should take into account factors such as cultural issues that might modify the patient's desire to receive such information.<br><br>In most cases, the psychiatrist will have an explicit discussion with family members regarding the diagnosis, prognosis, and treatment options, adapted to the unique concerns of the patient and family.<br><br>It is important to educate the patient and family about the range of symptoms that could develop in the current stage of dementia or that may develop in the future. This education allows them to plan for the future and to recognize emergent symptoms that should be brought to medical attention.<br><br>By treating these symptoms,	The patient and family may be troubled and fearful about these symptoms, and it may be helpful to reassure them that the symptoms are part of the illness and are often treatable.<br><br>Severely and Profoundly Impaired Patients<br>Families are often struggling with a combined sense of burden and loss and may benefit from a frank exploration of these feelings and any associated resentment or feelings of guilt.			

		educating family caregivers, and providing them with alternative strategies to deal with the patient's disruptive behaviors, the psychiatrist can help to minimize the caregivers' negative reactions to the patient's behavior.<br><br>Support Families During Decisions About Institutionalization<br>The psychiatrist can be a valuable resource in informing families about the available options and helping them evaluate and anticipate their needs in the context of their values, priorities, and other responsibilities.<br><br>The question of referral to a long-term-care facility should be raised well before it becomes an immediate necessity so that families who wish to pursue this option have time to select and apply for a suitable facility, plan for financing long-term care, and make needed emotional adjustments.<br><br>At each stage of the illness, the psychiatrist should be vigilant for cognitive and noncognitive symptoms likely to be present and should help the patient and family anticipate future symptoms. The family may also benefit from reminders to plan for the care likely to be necessary				

		at later stages.<br><br>Treatments for Psychosis and Agitation<br>This weighing of risks also includes consideration of the evidence supporting the efficacy of the agent in question, the patient's overall medical condition, and the evidence of risk and benefit of any potential treatment alternatives, followed by documentation of the reasons for using the medication and the fact that a discussion has taken place with the patient or caregiver.				
United States 2006 (Fillit et al., 2006)	--	Office based screening should also be initiated when a caregiver, patient, medical office employee, case manager, or assisted living facility staff notes the presence of cognitive impairment, such as memory loss or confusion, regardless of the patient's age.<br><br>Clinicians can obtain information from caregivers about behavioral issues, such as anxiety, apathy, hallucinations, depression, sleep disturbance, and other problems.<br><br>Patients and caregivers should be counseled with regard to "realistic" expectations of antidementia pharmacologic treatment.	--	--	--	It is the consensus panel's recommendation that best practice would identify patients in the early or mild stage of the disease, when pharmacotherapy and care management could preserve cognition and function in patients for the longest period, in the most independent state.<br><br>Alzheimer's disease care management should also include referrals for discussions of advanced directives, proxy assignment, and durable power of attorney, as well as financial planning for long-term care and medical assistance, as needed.

		Counseling caregivers with regard to expectations of antidementia therapy based on the results of clinical trials is critical to appropriate therapy in clinical practice.<br><br>Geriatric care management and counseling should be provided to all patients with a diagnosis of Alzheimer's disease and to their caregivers. Due to the complex medical and care management issues of dementia, the panel recommended that counseling and geriatric care management should be provided to all patients who have been diagnosed with ADRDs and to their caregivers. Physicians (particularly primary care physicians), other provider MCO medical management programs, and public service organizations, such as the Alzheimer's Association, can provide such counseling.				
Scotland 2006 (Scottish Intercolle giate Guidelines Network, 2006)	--	As a person with dementia may not be able to give a fully accurate history a relative or carer should also be interviewed.<br><br>The Informant Questionnaire on Cognitive Decline in the Elderly (IQCODE; see Annex 8) is a short questionnaire filled out by someone who knows the patient and can be an adjunct to direct cognitive testing.<br>A questionnaire, such as the IQCODE, completed by a relative	Practitioners involved in assessing and diagnosing dementia are responsible for what people know of their diagnosis, or whether they know their diagnosis at all. Diagnosis disclosure can	--	The ultimate judgement must be made by the appropriate healthcare professional(s) responsible for clinical decisions regarding a particular clinical procedure or treatment plan. This judgement should only be arrived at following discussion of the options with the patient, family and carers, covering the	Caregivers should receive comprehensive training on interventions that are effective for people with dementia.<br><br>Cognitive stimulation should be offered to individuals with dementia. Cognitive stimulation training can be carried out at home by a caregiver, with no risk to the person with dementia and with

		or friend may be used in the diagnosis of dementia.<br><br>There is a consensus that both people with dementia and their carers are entitled to receive relevant information<br><br>Information should not only include issues considered relevant by clinicians, but be tailored to the needs of patients and carers<br><br>Patients and carers should be offered information tailored to the patient's perceived needs.<br><br>Good communication between healthcare professionals, patients and carers is essential.<br><br>Healthcare professionals should be aware that some people with dementia may not wish to know their diagnosis.<br>Whilst people have the right not to know their diagnosis, inappropriate withholding of the diagnosis, was a source of distress for some<br><br>Formal permission to disclose the diagnosis to carers should be sought<br><br>healthcare professionals should be aware that in some situations disclosure of a diagnosis of dementia may be inappropriate	lead to mixed reactions, such as shock, distress, ambivalence or confirmation of existing suspicions.		diagnostic and treatment choices available.<br><br>Healthcare professionals should be aware that many people with dementia can understand their diagnosis, receive information and be involved in decision making.<br><br>The wishes of the person with dementia should be upheld at all times	minimal training/education of the carer.<br><br>Key areas to be considered for implementation are development of caregiver training programmes.<br><br>Caregivers should receive comprehensive training on interventions that are effective for people with dementia. Training of carers can take a variety of forms with the most common types being one to one observation by a community psychiatric nurse (CPN) and group training. The availability of such training varies across Scotland. It is likely that this recommendation would have some impact on CPN time.<br><br>Key areas to consider for audit are are complex caregiver intervention programmes being developed and implemented appropriately?

		The diagnosis of dementia should be given by a healthcare professional skilled in communication or counselling.<br><br>Patients and carers should be provided with information about the services and interventions available to them at all stages of the patient's journey of care.<br><br>Information should be offered to patients and carers in advance of the next stage of the illness.<br><br>Methods of disseminating information which may be appropriate for people with dementia and their carers include:<br>• written information<br>• individual education programmes<br>• group education programmes<br>• counselling<br>• telemedicine service<br>• communication workshops<br>• cognitive behaviour therapy (CBT)<br>• stress management<br>• combinations of the above.				

Additional File 3. Data extracted on dementia-specific aspects of person-centred care

Guideline	Sex/Gender	Intersectional factors	Dignity	Quality of life
Canada 2020 (Ismail et al., 2020)	--	--	--	--

United Kingdom 2018 (National Institute for Health and Care Excellence, 2018)	Service providers should design services to be accessible to as many people living with dementia as possible, including:<br>• people who have other responsibilities (such as work, children or being a carer themselves)<br><br>Care and support providers should provide all staff with training in person centred and outcome-focused care for people living with dementia, which should include:<br>• respecting the person's individual identity, sexuality and culture	Service providers should design services to be accessible to as many people living with dementia as possible, including:<br>• people who do not have a carer or whose carer cannot support them on their own<br>• people who do not have access to affordable transport, or find transport difficult to use<br>• people who have other responsibilities (such as work, children or being a carer themselves)<br>• people with learning disabilities, sensory impairment (such as sight or hearing loss) or physical disabilities<br>• people who may be less likely to access health and social care services, such as people from black, Asian and minority ethnic groups.<br><br>Healthcare professionals should also be mindful of the need to secure equality of access to treatment for patients from different ethnic groups, in particular those from different cultural backgrounds.<br><br>When assessing the severity of Alzheimer's disease and the need for treatment, healthcare professionals should not rely solely on cognition scores in circumstances in which it would be inappropriate to do so. These include:<br>• if the cognition score is not, or is not by itself, a clinically appropriate tool for assessing the severity of that patient's	Fear of stigma prevented carers and people living with dementia from seeking help.	Offer a range of activities to promote wellbeing that are tailored to the person's preferences.<br><br>Consider cognitive rehabilitation or occupational therapy to support functional ability in people living with mild to moderate dementia.<br><br>For people living with dementia who have sleep problems, consider a personalized multicomponent sleep management approach that includes sleep hygiene education, exposure to daylight, exercise and personalized activities.<br><br>When thinking about admission to hospital for a person living with dementia, take into account:<br>the value of keeping them in a familiar environment.<br><br>Offer carers of people living with dementia a psychoeducation and skills training intervention that includes:<br>• advice on planning enjoyable and meaningful activities to do with the person they care for<br>• available at a location they can get to easily

		dementia because of the patient's learning difficulties or other disabilities (for example, sensory impairments), linguistic or other communication difficulties or level of education or<br>• if it is not possible to apply the tool in a language in which the patient is sufficiently fluent for it to be appropriate for assessing the severity of dementia or<br>• if there are other similar reasons why using a cognition score, or the score alone, would be inappropriate for assessing the severity of dementia.<br>In such cases healthcare professionals should determine the need for initiation or continuation of treatment by using another appropriate method of assessment.<br><br>Care and support providers should provide all staff with training in person centred and outcome-focused care for people living with dementia, which should include:<br>• respecting the person's individual identity, sexuality and culture		
Canada 2017 (Toward Optimum Practice, 2017)	--	Patient-centred and culturally sensitive care should be provided at all times.	--	--
Australia 2016 (Guideline Adaptation Committee	--	<u>Language/Culture</u><br>People with dementia who develop behavioural and psychological symptoms should be offered a comprehensive assessment at an early opportunity by a professional skilled in	As people with dementia are vulnerable to abuse and neglect, all health and aged care staff supporting	Improving quality of life, maintaining function and maximizing comfort are appropriate for people living with dementia throughout the disease trajectory, with the emphasis on particular goals changing over time.

, 2016)		symptom assessment and management. This should involve their carer(s) and families as appropriate, and include the influence of religious and spiritual beliefs and cultural norms<br><br>The Rowland Universal Dementia Assessment Scale (RUDAS) should be considered for assessing cognition in CALD populations.<br><br>If language or culture is a barrier to accessing or understanding services, treatment and care, health and aged care professionals should provide the person with dementia and/or their carer(s) and family with:<br>• information in the preferred language and in an accessible format<br>• professional interpreters<br>• interventions in the preferred language.<br><br>Health and aged care services need to recognize and be responsive to the cultural and linguistic needs of CALD people living with dementia, their carer(s) and families. Services should utilize a range of communication tools, including working with bilingual bicultural staff or professional interpreters across the whole service pathway, particularly during assessment, when communicating the diagnosis and gaining consent.<br><br>CALD carers and families should receive support, education and information, through partnerships with ethno-specific and mainstream agencies and	people with dementia should receive information and training about how to prevent and manage suspected abuse.	Health and aged care professionals should inform the person with dementia, their carer(s) and family about advocacy services and voluntary support, and should encourage their use. If required, such services should be available for both the person with dementia and their carer(s) and family independently of each other.<br><br>Organizations in primary, secondary and tertiary care settings should consider the needs of people with dementia when designing health and aged care services and facilities. In particular, services should be structured to complement existing services in the local area.<br><br>People with dementia living in the community should be offered occupational therapy interventions which should include: environmental assessment and modification to aid independent functioning; prescription of assistive technology; and tailored intervention to promote independence in activities of daily living which may involve problem solving, task simplification and education and skills training for their carer(s) and family.

		they should be delivered by bilingual, bicultural workers in the field.<br><br>Clinical cognitive assessment in those with suspected dementia should include examination using an instrument with established reliability and validity. Health and aged care professionals should take full account of other factors known to affect performance, including age, educational level, non-English speaking background, prior level of functioning, aphasia, hearing or visual impairments, psychiatric illness or physical/ neurological problems when interpreting scores.<br><br><u>Age</u><br>People with younger onset dementia have unique needs; organizations should tailor their services in order to ensure that they are age appropriate and address the needs of the person with younger onset dementia and their carer(s) and family.<br><br><u>Indigenous</u><br>Consultation with Indigenous community representatives and the local Indigenous medical service should occur in the development, implementation and review of any initiative intended for Indigenous communities. The formation of an Indigenous advisory committee or consultation with an existing committee ensures ongoing collaboration. Where appropriate, groups should consult with Alzheimer's Australia's National Aboriginal and Torres Strait Islander		

		Dementia Advisory Group and State or Territory Indigenous peak health bodies.<br><br>Health and aged care services working to improve the health and care of Indigenous Australians living with dementia should be culturally sensitive and informed and utilize translators and/or cultural interpreters where necessary, particularly during assessment, when communicating the diagnosis and gaining consent.<br><br>Health and aged care professionals should consult with family and Indigenous community representatives when developing a culturally appropriate care plan. A case manager (who may be an Indigenous community-based staff member) can assist with accessing and coordinating services required and advocating for the person with dementia.<br><br>The Kimberley Indigenous Cognitive Assessment (KICA-Cog) or KICA-Screen tool is recommended for use with remote living Indigenous Australians for whom the use of alternative cognitive assessment tools is not considered appropriate. The modified KICA (mKICA) is recommended as an alternative to the Mini Mental State Exam (MMSE) in urban and rural Indigenous Australian populations when illiteracy, language or cultural considerations deem it appropriate.		
Canada 2016 (The Governme	--	The patient's cultural and educational background need to be considered when administering and interpreting	Consider the following general care and	--

nt of British Colombia, 2016)		results of assessment tools<br><br>The assessment and management of cognitive impairment in diverse individuals can be challenging for several reasons:<br>• Communication difficulties, cultural factors, low education and literacy impact formal cognitive screening, with poor interrater reliability – use interpreter services to assist in more accurate patient screening and assessment;<br>• Dementia symptoms may be unfamiliar or viewed as part of the aging process, and there may be stigma to mental health issues, resulting in diagnosis delay – provide culturally sensitive patient information on dementia to patients and families;<br>• Language barriers may result in a lack of awareness of community supports – provide Guide for Patients and Caregivers;<br>• Families may share caregiver responsibilities by rotating the residence of the patient amongst family members – this is generally discouraged as it confuses the patient with dementia and complicates the provision of services between the staff of many agencies and the extended family. One familiar, safe and secure environment is encouraged.	supplementary supports for patients: Self-Neglect, Neglect and Abuse<br>Help assess patient abuse/neglect using the Re: Act Adult Abuse and Neglect Response Flow Sheet and Assessment Guide	
Singapore 2013 (Nagaendran et al., 2013)	--	Financial difficulties should be asked for and if problems are identified, families can be referred to a social worker	Nevertheless, person-centred care that emphasizes the dignity and autonomy of the patient, should be	Environmental design features may be incorporated in care facilities to reduce behavioural and psychological symptoms of dementia (BPSD) in people with dementia (pg 52).<br><br>Environmental design features may be incorporated

			upheld at all stages irrespective of the patient's functional status.<br><br>CPR can be burdensome, undignified and carries the risk of harm. For these reasons, it should not be routinely performed for persons with advanced dementia.	in care facilities to reduce behavioural and psychological symptoms of dementia (BPSD) in people with dementia<br><br>Therapeutic goals and treatment options that are coherent and appropriate to patient's functional status should be individually tailored and adjusted at different stages of dementia, with an emphasis on quality for life of the patient, as well as the caregiver.<br><br>5. If the patient lacks adequate decision making capacity, the ethical imperative switches to one that aims to protect the patient from his or her own harmful decisions or actions. Patients must not be under-treated nor forced to receive inappropriate treatment just because they lack decision making capacity or legally appointed guardian(s). Consideration of the patient's functional status and quality of life is vital in making treatment decisions for the patient.<br><br>6.7 living alone<br>Considerations should also be given to the potential negative social and physical impact of moving from a familiar environment to institutional care<br><br>5. The final objective is to provide the patient with dementia with a safe, familiar and comfortable living environment, and to avoid premature institutionalization.<br><br>Patients with YOD have special requirements and will benefit from specialist multidisciplinary care. A multidisciplinary approach to YOD will allow early diagnosis and treatment of reversible syndromes while for non-reversible neurodegenerative diseases, early counselling and provision of support services would improve the quality of life of patients and their caregivers

				Integral to a comprehensive management plan is the referral of persons with dementia to appropriate community resources. This assists in providing care and improving quality of life of both the person with dementia and their caregivers
Czech Republic 2010 (Hort et al., 2010)	--	The most widely used screening test (I) is the Mini-Mental State Examination (MMSE), which standard cut-off score (24) should be increased to 27 in highly educated individuals and lowered in patients whose native tongue is another language or with low education.<br><br>Differences among ethnic, cultural, and religious groups may influence how and what disclosure occurs.	--	Careful assessment for any co-morbidities and consideration given to other services that may be required including social services, mental stimulation, occupational therapy, physiotherapy, speech and language therapy (IV). Occupational therapy can benefit patients' daily functioning and reduce the need for informal care.<br><br>Driving, medico-legal issues and the need for other support services should be considered (good practice point).<br><br>Cognitive stimulation or rehabilitation may be considered in patients with mild to moderate AD (good practice point). Occupational therapy can improve patients functioning and reduce need for informal care (Level B).
Spain 2010 (Ministry of Health Social Services and Equality, 2010)	--	--	Despite the existence of the AFA and the consensus about their efficiency and effectiveness, less than 30% of the families affected ever get in contact with the relative AFA to get information or any other type of resources. There are many different reasons, including the lack of information about their existence, the	Psychological support and psychoeducational interventions geared towards families with people with dementia are recommendable, in order to favour their task of caring, reduce the care burden and improve the quality of life.<br>The systematic assessment of the presence of BPSD is recommended as it is a fundamental component of the clinical picture, it appears very frequently, it is difficult to manage, it has an impact on the quality of life and is a frequent reason for urgent care, family claudication and institutionalization.<br>Whatever their location, it is recommendable for patients with advanced or terminal dementia to receive palliative measures to improve their comfort, suffering and quality of life, insofar as this is possible.<br>Donepezil showed no benefit in quality of life of the

			refusal of the patient and/or family to do so for reasons of confidentiality, culture, education or due to rejection on considering it to be marginal and/or with a risk of being stigmatized.<br>The dissemination of knowledge on dementia through the media must be done adequately and in a way that is understandable for the different groups that make up society.647Currently the media references about people affected by dementia are routine,648 therefore the subject must be dealt with normally and with the necessary sensitivity to prevent dramatizing or stigmatizing people who suffer the problem, always with due respect to the privacy and dignity of the person affected and of their environment.	patient either after 12 or after 24 weeks. The quality of life has not been assessed with other drugs.<br>There is no evidence to recommend treatment with ACE inhibitors to improve the quality of life of patients with AD.<br>There is no evidence in VD of the influence of ACE inhibitors and/or memantine on the quality of life of patients and of their caregivers.<br>The aim of PC is to relieve physical and psychological suffering, promote quality of life and consider death as a natural process. Psychosocial and spiritual aspects must be integrated, giving support to relatives throughout the entire process. They must be introduced after the diagnosis and be intensified throughout the evolution

Malaysia 2009 (Ministry of health, Malaysia, 2009)	--	All tests need to be validated for the culture, language and educational level of the population.<br><br>Appropriate tools that can be used for detailed screening include MMSE or ECAQ and GDS-4 to be used.<br><br>The ECAQ is specifically designed to assess cognition among the elderly population in developing countries, taking into account the cultural difference and the relatively low literacy rate	--	Recommendation. Environmental modification is helpful but needs to be individualized to the person and the degree of impairment, preferably after occupational therapist assessment.<br><br>ENVIRONMENTAL DESIGNS APPROPRIATE FOR PEOPLE WITH DEMENTIA General Recommendations<br>A) Institution Unit size:<br>• Give consideration to the size of units, mix of residents and the skill mix of staff to ensure that the environment is supportive and therapeutic.<br>• Smaller units are associated with gains that include less anxiety and depression, greater mobility, increase in supervision and interaction between caregiver and residents, higher motor functioning, improve or maintenance of ADL's and lowers level of strain and better attitude to dementia care.<br>Dining:<br>• Food not served in tray and served in a common dining room.<br>Other recommendations:<br>• Simulated home environment/show rooms in the hospital/community settings.<br>• Usual care consists of education materials and booklet describing home environment safety tips.<br>• Floor with coarse-textured coverings, shining, with sharp colour contrast and highly patterned surfaces can be misinterpreted as change in level by people with impaired depth perception.<br>B) Domestic setting<br>• When organizing living arrangements and/or care homes for PWD, must ensure that built environment are enabling and aid orientation. Attention should be given to lighting, colour schemes, floor coverings, assistive technology, signage, garden design and the access to and safety of the external environment.<br>• Incorporate outdoor areas with therapeutic design features.

				• Using covers over fire exit bars and door knobs helps to reduce unwanted exiting.<br>• Tactile way finding cues, good lighting and windows allowing daylight to enter, view of external landmarks may help PWD to find their way around the indoor environment.<br>• Colors may also be used to assist with orientation.<br>• Highly visible toilets may potentially reduce level of incontinence.<br>• Providing moderate level of environmental stimulation is also recommended such as murals on walls.<br>• Wandering paths are recommended.
United States 2012 (Amante et al., 2012)	--	Comparison of cognitive assessment tools<br>Mini-Cog, MMSE, MoCA, GPCOG<br>1. Interpret scores taking into full account the factors known to affect performance, including educational level, skills, prior level of functioning and attainment, language and sensory impairment, psychiatric illness, and physical /neurological problems.<br>MMSE, MoCA<br>2. Score may need to be adjusted to account for level of education.	Goals of treatment for dementia- Late-stage Dementia<br>Preserve safety, comfort, and dignity	Encourage Claire to maintain a healthy diet, regular physical activity, and her and Cliff's social connections. Also talk about keeping an eye on her blood pressure. In addition, she might try taking up new and challenging mental activities, such as a language or musical instrument. (Note that commercial products marketed at seniors for retaining memory remain unproven.)<br><br>Goals of treatment for dementia-Mild cognitive impairment/ memory loss<br>Maintain function, safety, and independence.<br><br>Goals of treatment for dementia- Early-stage dementia<br>Maintain function and independence while preserving safety.<br><br>Lifestyle modifications and nonpharmacologic options. The following studied interventions have resulted in benefits to cognitive function:<br>• Exercise—Group Health currently offers both the Enhance Fitness and Silver Sneakers fitness programs (www.ghc.org/classesAndEvents/seniorFitness.jhtml). Many other exercise programs targeting seniors are offered in the community.

				• Activity and socialization—Introducing pleasant activities daily can improve mood and increase quality of life. Consider ways to increase socialization, including day treatment and occupational therapy programs.
Australia 2008 (Abbey et al., 2008)	--	The principles that underlie the National Framework for Action on Dementia and inform the development of these Guidelines and Pathways are:<br>All people with dementia, their carers and families receive appropriate services that respond to their social, cultural or economic background or location<br><br>Equitable access to dementia related information, support and services should be available to people with dementia, their carers and family members, including those in special circumstances such as people of different cultural backgrounds, sexual orientation and those living in rural and remote areas<br><br>People with dementia and their carers may find that their financial status is affected (17) and the benefits available should be discussed with the carer. Health professionals need to be aware of the financial impact of caring for a person with dementia at home, discuss the benefits available and refer to appropriate organizations when necessary.<br><br>Factors such as visual impairment,	Guideline<br>People living with dementia are at risk of abuse and health professionals should be aware of this possibility.	The principles that underlie the National Framework for Action on Dementia and inform the development of these Guidelines and Pathways are (4):<br>• People with dementia are valued and respected. Their right to dignity and quality of life is supported<br>• Communities play an important role in the quality of life of people with dementia, their carers and families<br><br>Functional assessment<br>A home visit is useful to obtain a history and to assess the safety and quality of the environment in which the person lives (22), including risk of falls<br><br>The Alzheimer's Australia website offers information about the physical environment which might be of assistance in preventing falls<br>Funded services may be able to assist through occupational therapy intervention and arranging home modification<br><br>Advanced Phase<br>In this phase health professionals are responsible for:<br>• Ensuring that the person with dementia and their carers have access to appropriate services that provide a palliative approach<br>• Reviewing goals of care to promote comfort, quality of life and dignity<br>• Providing management strategies for maximizing comfort

		sensory impairment and physical disability need to be assessed and considered in selecting the mental status tests to be implemented.<br><br>An interpreter may be required for people who are hearing impaired or of a CALD background<br><br>There is a consensus among health professionals that favours disclosure of diagnosis; however health professionals should be aware that some people with dementia and/or their carers may not wish to know their diagnosis. This may be particularly pertinent in relation to Indigenous people and people who are from CALD backgrounds. For these groups it may be culturally insensitive or inappropriate to disclose a diagnosis.<br><br><u>Indigenous</u><br>The KICACog (19) is recommended by the DOMS Project as a culturally appropriate method of assessing dementia in Indigenous peoples over the age of 45 years who are living in rural and remote areas of Australia<br><br>The shortened version, the KICA-Screen (19), consisting of ten subject cognition questions used in conjunction with an eight item informant questionnaire, the KICA-Carer (19), is recommended as a screening tool for use in Primary Care and General Practice to determine the presence of cognitive impairment in rural and remote Indigenous peoples.<br><br>Other aspects, particularly in the		

		Australian context are language and cultural appropriateness. Instruments recommended for cognitive assessment include:<br>The KICA-Cog for Indigenous Australians who are aged over 45 years and from rural and remote areas.<br><br>The Rowland Universal Dementia Assessment Scale (RUDAS) (31) has recently been developed in Australia and is suitable across most cultures. While limited testing of this scale has been undertaken early indications are that the tool is valid and reliable. It is recommended as a short cognitive screening tool for the assessment of dementia in multicultural population groups by primary health care workers.		
United States 2008 (California Workgroup on Guidelines for Alzheimer's Disease Manageme nt, 2008)	--	The following questions provide a framework for conducting the cultural assessment recommended in this section:<br>1. What is the patient's and family's preferred (i.e., most comfortable) language for communicating with the PCP? If not English, is there a bilingual person available to assist?<br>2. How "acculturated" are the patient and family? How well equipped are they to manage clinical and other service referrals that the PCP may suggest?<br>3. How do members of the patient's cultural group relate to each other, to those in authority (e.g., PCPs and staff members), or to strangers?<br>4. What sources of cultural information are available to help the PCP make this assessment (e.g., patient self-report, reports of family members or other	Conduct and document an assessment and monitor changes in: abuse and/or neglect<br><br>Elder Abuse. Monitor for evidence of and report all suspicions of abuse (physical, sexual, financial, neglect, isolation, abandonment, abduction) to adult Protective services, long Term Care ombudsman, or the local police department, as required by law.	Nevertheless, PCPs should be able to provide or recommend a wide variety of services beyond medical management of Alzheimer's Disease and comorbid conditions, including recommendations regarding psychosocial issues, assistance to families and caregivers, and referral to legal and financial resources in the community. Many specialized services are available to help patients and families manage these aspects of AD, such as adult day services, respite care, and skilled nursing care, as well as helplines and outreach services operated by the Alzheimer's Association, Area Agencies on Aging, Councils on Aging, and Caregiver Resource Centers<br><br>Conduct and document an assessment and monitor changes in:<br>Living arrangement, safety, care needs<br><br>Develop and implement an ongoing treatment plan with defined goals.

		caregivers, other service providers, direct observation by the PCP)?<br><br>Identify the patient's and family's culture, values, primary language, literacy level, and decision-making process.<br><br>PCPs need to tap into underlying belief systems regarding Alzheimer's Disease and other comorbid conditions. This underlying world view and accompanying normative expectations are often expressed in terms of "folk understandings" which may influence the way in which people from diverse cultures receive and act on the information and directions provided by the PCP.<br><br>Functional assessment includes evaluation of physical, psychological, and socioeconomic domains.<br><br>All of these instruments have been validated and some are available in languages other than English (e.g., Spanish, Tagalog, Cantonese). Regardless of the instrument used, the PCP needs to consider the effect that literacy level and language may have on cognitive screening test scores.<br><br>Caregiver assessment should be multidimensional, reflect culturally competent practice, and be updated periodically<br><br>The PCP must be culturally competent for appropriate and most effective	Conduct and document an assessment and monitor changes in: Living arrangement, safety, care needs, and abuse and/or neglect.<br><br>Abuse and Neglect. With respect to the patient, simple questions such as: "Are you afraid of anyone? Is anyone stealing from you? Has anyone hurt you?" are easy ways to screen for abuse.<br><br>In addition, the most important care recipient characteristics to look for in assessing for potential abuse are:<br>• Problems with short-term memory;<br>• Psychiatric diagnosis;<br>• Alcohol abuse;<br>• Difficulty interacting with others;<br>• Self-reported conflict with family members and friends;<br>• Feelings of	Discuss with patient and family: Referral to early-stage groups or adult day services for appropriate structured activities, such as physical exercise and recreation<br><br>Develop Treatment Plan. Discuss with patient and family:<br>Referral to early-stage groups or adult day services for appropriate structured activities, such as physical exercise and recreation<br><br>Treat behavioral symptoms and mood disorders using:<br>Non-pharmacologic approaches, such as environmental modification<br><br>Conduct and document an assessment and monitor changes in:<br>Living arrangement, safety, care needs<br><br>Assessment of a patient's living environment can identify environmental supports that may be needed to maximize function, ensure safety, and minimize caregiver stress.<br><br>Assessment of a patient's living environment may help identify retained abilities and things the individual is able to do within a familiar setting. It can also aid in identifying environmental supports that may be needed to maximize function, ensure safety, and minimize caregiver stress.<br><br>Develop and implement an ongoing treatment plan with defined goals. Discuss with patient and family: Referral to early-stage groups or adult day services for appropriate structured activities, such as physical exercise and recreation.<br><br>The PCP is in a unique and influential position to

		evaluation and treatment of Alzheimer's Disease<br><br>PCPs need to be sensitive to the preferred language of the patient and family, which may determine service linkage and adherence outcomes. In ethnically diverse populations, bilingual families may have quite different service engagement outcomes than monolinguals.<br><br>The PCP should consult with the primary caregiver to identify beliefs about health and aging, learn about cultural taboos (e.g., direct eye contact), determine the language or dialect spoken by the patient and the patient's family, and utilize bilingual, bicultural health care providers as appropriate.<br><br>PCPs should be aware that paper and pencil tests and forms may not work well with the diverse populations they treat, if basic literacy is not present, even when such forms are in the persons' (or groups') native language. Therefore, PCPs should consider both culturally as well as literacy-appropriate assessment tool.<br><br>Cognitive screening tools such as the Cognitive Abilities Screening Instrument (CASI), which are relatively unaffected by cross cultural bias and education level, may be administered to persons of both high and low education and are especially useful when working with ethnically diverse populations. The same concerns extend to printed	loneliness; and<br>• Inadequate or unreliable support system (Shugarman et al., 2003).<br>It is recommended that patients exhibiting three of the seven predictors of potential abuse be targeted for further investigation, although fewer "triggers" also may signal a strong need for preventive measures such as additional support services<br><br>Because timely referrals to support services may help mitigate or eliminate circumstances associated with abuse and neglect, thorough assessment and monitoring by the PCP is essential to the safety of both patient and caregiver.<br><br>Recommendation: Conduct and document an assessment and monitor changes in living arrangements,	direct the Alzheimer's Disease patient and family to available resources that may assist in care provision and improve the quality of life of both patient and caregiver<br><br>Recommendations: Develop and implement an ongoing treatment plan with defined goals. Discuss with patient and family referrals to early-stage groups or adult day services for appropriate structured activities, such as physical exercise and recreation.<br><br>non-pharmacologic interventions may begin with a modification of the patient's environment and routine (see Table T6 in this section). Special attention should be paid to the triggers of the problem behavior to select effective, individualized interventions. The goal is often reduction or modification of the behavior rather than total elimination. The PCP should encourage the establishment of an exercise routine for the patient, to maintain ambulation and improve patient behavior and mood<br><br>Checklist for Early-Stage Care<br>Recommend the following non-pharmacological interventions (preferably in combination) to protect and promote continuing functioning, assist with independence, and maintain cognitive health:<br>1. Physical exercise, preferably aerobic exercises if tolerated (or less-strenuous exercises that promote strength, balance, and coordination, such as tai Chi);<br>5. Programs to improve sleep, such as niTe-alzheimer's Disease<br><br>Factors to Consider in Planning for End-of-Life Care<br>• Maximize options for comfort care through hospice referral<br>• avoid futile care and prolongation of the dying process

		information about Alzheimer's Disease that may be provided to patients and their families. The content may require a literacy level that is too high for the persons receiving it; thus alternatives, such as more pictorially presented materials, may need to be considered<br><br>In making referrals to adult day services or any other community-based services, it is essential that recommendations be individualized to the particular patient's and/ or family's needs. It is particularly important that PCPs attend to cultural and language issues (see Assessment section). Referrals must be made to services that are consistent with cultural values and to organizations that can accommodate the needs (e.g., language) of individuals from different ethnic backgrounds.<br><br>Factors to Consider in Planning for End-of-Life Care: Discuss the intensity of care and other end-of-life care decisions with the Alzheimer's Disease patient and involved family members while respecting their cultural preferences.<br><br>It is especially important for there to be legal documentation of a patient's wishes because certain cultures rely on "fictive kin" (non-blood relatives who are considered family) to make medical treatment decisions. Because the law does not recognize non-blood relatives, PCPs may inadvertently discount them, while the family may not only value the input of non-blood relatives but actually depend on them with regard to	safety, care needs, and abuse and/or neglect.<br><br>The following questions provide a framework for conducting the cultural assessment recommended in this section:<br>What other, non-cultural elements may skew the PCP's understanding of cultural factors influencing treatment outcomes (e.g., stereotyping)?<br><br>The PCP should monitor for abuse as well as offer interventions to the patient and caregiver through medical treatment and referrals to community agencies.<br><br>Elder Abuse: Monitor for evidence of and report all suspicions of abuse (physical, sexual, financial, neglect, isolation, abandonment, abduction) to Adult Protective Services, Long Term Care	As the end of the patient's life approaches, the PCP needs to present care options that maximize comfort and other potential benefits while avoiding futile treatments that may not provide comfort and may actually prolong the dying process.<br><br>The PCP can make clinical evaluations concerning the capacity of the patient and, when the patient is no longer legally capable of making particular kinds of decisions, should provide guidance to families, attorneys, and courts to assist the patient to live within boundaries that constitute the least restrictive alternatives

		important decisions on treatment and other matters<br><br>The PCP should refer the family to the Alzheimer's Association, Caregiver Resource Center, or other community organization to identify appropriate local legal resources with experience in dealing with non-English-speaking and/ or low-literacy populations.	Ombudsman, or the local police department, as required by law.<br><br>There is evidence that certain patient attributes (e.g., cognitive or functional impairment and physical dependence) may be predictors of risk for violence in Alzheimer's Disease families, and that caregiver depression and living arrangements as well as other factors are associated with both verbal and physical abuse. Thus, the health care team has the responsibility to monitor and intervene where required<br><br>The obligation of the PCP is to provide support and referrals to both patient and caregiver and to intervene appropriately if abuse is suspected	
United States 2007	There are more women with dementia,	- A social service referral may be helpful for some families to help with	Agitation and Aggression	Recommended assessments include evaluation of suicidality, dangerousness to self and others, and

(Rabins et al., 2007)	partly because of greater longevity, but also because Alzheimer's<br>disease is more prevalent among women for reasons that are not known.<br>In addition, because of their<br>greater life expectancy (and tendency to marry men older<br>than themselves), women with dementia are more likely to have an adult child rather than a spouse as caregiver.<br>Unlike an elderly spouse caregiver, who is more likely to<br>be retired, adult child caregivers (most often daughters or<br>daughters-in-law) are more likely to have jobs outside the<br>home and/or to be raising children. These additional caregiver responsibilities may contribute to earlier institutionalization<br>for elderly women with dementia	transportation arrangements and costs. A patient with more complex financial issues should be referred to an attorney or financial planner to establish appropriate trusts, plan for transfer of assets, and make other financial arrangements.<br><br>Another critical demographic factor affecting the care of patients with dementia is social support. The availability of a spouse, adult child, or other loved one with the physical and emotional ability to supervise and care for the patient, communicate with treating physicians, and otherwise coordinate care may influence the patient's quality of life as well as the need for institutionalization. In addition, a social network of friends, neighbors, and community may play a key role in supporting the patient and primary caregivers. Spiritual supports and religious beliefs have been shown to have positive benefits for caregivers' well-being. These findings should be taken into account in assessment and treatment planning.<br><br>Resource availability varies widely by geographic region and socioeconomic status. This issue should be considered in all treatment decisions but has a particular impact on decisions about long-term care. A referral to the local chapter of the Alzheimer's Association or to a social worker or another individual knowledgeable about local resources, treatment centers, and	Whatever the intervention, it is critical to match the level of demand on the patient with his or her current capacity, avoiding both infantilization and frustration Nonetheless, principles of humane care as well as federal regulations support minimizing restraint use as much as possible.<br><br>Abuse and Neglect<br>The psychiatrist should be alert to the possibility of elder abuse, financial exploitation, and neglect. Individuals with dementia are at particular risk for abuse because of their limited ability to protest, their lack of comprehension, and the significant demands and emotional strain on caregivers. Patients whose caregivers appear angry or frustrated may be at even higher risk. Any concern, especially one raised by the	the potential for aggression, as well as evaluation of living conditions, safety of the environment, adequacy of supervision, and evidence of neglect or abuse.<br><br>The need for restraints can be decreased by environmental changes that decrease the risk of falls or wandering and by careful assessment and treatment of possible causes of agitation.<br><br>Mildly Impaired Patients:<br>At the early stages of a dementing illness, patients and their families are often dealing with acceptance of the illness and recognition of associated limitations. They may benefit from pragmatic suggestions for how to cope with these limitations (e.g., making lists, using a calendar, avoiding overwhelming situations such as certain childcare responsibilities). Patients may benefit from referral to health promotion activities and recreation clubs. It may be helpful to identify specific impairments and highlight remaining abilities.<br><br>Implementation of Psychosocial Treatments<br>In addition, some patients may benefit from more specific psychosocial interventions. These more specific psychosocial treatments for dementia can be divided into four broad groups: behavior oriented, emotion oriented, cognition oriented, and stimulation oriented. Although these treatment approaches differ in philosophy, focus, and methods, they share the broadly overlapping goals of improving quality of life and maximizing function in the context of existing deficits

		Medicaid laws can be important in helping families find local treatment options that fit their needs and budget.	patient, must be thoroughly evaluated. Stimulation-oriented treatments, such as recreational activity, art therapy, music therapy, and pet therapy, along with other formal and informal means of maximizing pleasurable activities for patients, have modest support from clinical trials for improving behavior, mood, and, to a lesser extent, function, and common sense supports their use as part of the humane care of patients.<br><br>Advise the Family to Address Financial and Legal Issues<br>In some instances, it may be a good idea to warn families about the vulnerability of individuals with dementia to unscrupulous individuals seeking “charitable” contributions, selling inappropriate goods,	

			or promoting sweepstakes. Clinicians should remain vigilant for evidence of exploitation of patients.	
United States 2006 (Fillit et al., 2006)	--	The panel recommended the following general guide-posts for identification of patients at various stages of the disease in clinical practice, using the Mini Mental State Examination (MMSE) scores: mild disease, 24- 20; moderate disease, 19 10; severe disease, <10; and profound disease, 0 or a score cannot be obtained. Some high-functioning people with MMSE scores >24 may also suffer mild dementia. These scores require an adjustment according to the patient's level of education.<br><br>Patients--particularly the frail and indigent elderly-- may either not fill prescriptions, or refill prescriptions and then skip or cut doses of necessary medication because of financial concerns. Therefore, the panel recommended that anti dementia therapy be provided to patients in a preferred formulary position to ensure appropriate utilization, compliance, and persistence.	--	Appropriate utilization of antidementia therapy and care management is vitally important in achieving quality of life and care for dementia patients and their caregivers, and for man aging the excess costs of Alzheimer's disease.<br><br>The panel acknowledged that, at this time, there are no conclusive, prospective, randomized studies demonstrating the value of screening for cognitive impairment in the elderly. Despite this, the panel determined that the impact of cognitive impairment on quality of care, quality of life, and costs of care, as well as the increasing prevalence of dementia with age, jus titles an age dependent, annual or biannual screening process, especially in people aged _>75 years (Table I). This recommendation is consistent with what others have recently proposed.<br><br>Appropriate utilization of antidementia therapy and care management is vitally important to achieving quality of life and care for dementia patients and their caregivers, and for managing the excess costs of Alzheimer's disease
Scotland 2006 (Scottish Intercollegiate Guidelines Network,	OESTROGEN<br>Dementia, particularly AD, is more common in postmenopausal women than any other population subgroup. Treatment with oestrogen has been proposed as a possible	--	--	Behaviour management may be used to reduce depression in people with dementia. Evidence suggests that reduction of repetitive verbalizations, management of aggression and management of eating behaviours in people with dementia have a positive effect on behaviour and well-being.<br><br>Multilevel behavioural management interventions

2006)	therapeutic agent for treatment of AD in women. There is evidence to suggest that oestrogen is ineffective for the prevention of cognitive decline in women with dementia. Oestrogen is not recommended for the treatment of associated symptoms in women with dementia.			may be more effective than individual interventions at improving behaviour and well-being in people with dementia.<br><br>Environmental design<br>Residential unit design, such as corridor configuration, can influence restlessness, anxiety and disorientation in institutionalized people with dementia. Given that people with dementia experience increasing memory impairment and cognitive decline it is important to have an environment that aids orientation.<br><br>Measures which should be considered when planning an environment for people with dementia include:<br>• incorporating small size units<br>• separating non-cognitively impaired residents from people with dementia<br>• offering respite care as a complement to home care<br>• relocating residents, when necessary, in intact units rather than individually<br>• incorporating non-institutional design throughout the facility and in dining rooms in particular<br>• moderating levels of stimulation<br>• incorporating higher light levels<br>• using covers over fire exit bars and door knobs to reduce unwanted exiting<br>• incorporating outdoor areas with therapeutic design features<br>• considering making toilets more visible to potentially reduce incontinence<br>• eliminating factors that increase stress when bathing.<br><br>For people with dementia, a combination of structured exercise and conversation may help maintain mobility

				Recreational activities should be introduced to people with dementia to enhance quality of life and well-being.<br>• Individualized activities adapted to maximize the person’s remaining abilities and based on previous interests may be more beneficial to people with dementia than generic activities.<br><br>Recreational activities should be introduced to people with dementia to enhance quality of life and well-being.<br><br>For people with dementia, a combination of structured exercise and conversation may help maintain mobility.
